# The mindful moms training: development of a mindfulness-based intervention to reduce stress and overeating during pregnancy

**DOI:** 10.1186/s12884-018-1757-6

**Published:** 2018-06-01

**Authors:** Cassandra Vieten, Barbara A. Laraia, Jean Kristeller, Nancy Adler, Kimberly Coleman-Phox, Nicole R. Bush, Helané Wahbeh, Larissa G. Duncan, Elissa Epel

**Affiliations:** 1 California Pacific Medical Center Research Institute, 475 Brannan Street, San Francisco, CA 94120 USA; 2 Institute of Noetic Sciences, 625 Second Street, # 200, Petaluma, CA 94952 USA; 3 Center for Health and Community, University of California, San Francisco, 3333 California St., Suite 465, Box 0844, San Francisco, CA 94143-0844 USA; 4 School of Public Health, University of California, Berkeley, 207-B University Hall, Berkeley, CA 94720-7360 USA; 5 Department of Psychology, Indiana State University, Terre Haute, Indiana, 47809 USA; 6 Department of Pediatrics, University of California, San Francisco, 550 16th Street, San Francisco, CA 94158 USA; 7 School of Human Ecology, University of Wisconsin-Madison, 1300 Linden Drive, Madison, WI 53706 USA

**Keywords:** Pregnancy, Obesity, Stress, Depression, Acceptance-based coping, Emotion regulation, Gestational weight gain, Behavioral intervention, Mindfulness, Mindful motherhood training

## Abstract

**Background:**

Pregnancy is a time of high risk for excessive weight gain, leading to health-related consequences for mothers and offspring. Theory-based obesity interventions that target proposed mechanisms of biobehavioral change are needed, in addition to simply providing nutritional and weight gain directives. Mindfulness training is hypothesized to reduce stress and non-homeostatic eating behaviors – or eating for reasons other than hunger or caloric need. We developed a mindfulness-based intervention for high-risk, low-income overweight pregnant women over a series of iterative waves using the Obesity-Related Behavioral Intervention Trials (ORBIT) model of intervention development, and tested its effects on stress and eating behaviors.

**Methods:**

Overweight pregnant women (*n* = 110) in their second trimester were enrolled in an 8-week group intervention. Feasibility, acceptability, and facilitator fidelity were assessed, as well as stress, depression and eating behaviors before and after the intervention. We also examined whether pre-to-post intervention changes in outcomes of well-being and eating behaviors were associated with changes in proposed mechanisms of mindfulness, acceptance, and emotion regulation.

**Results:**

Participants attended a mean of 5.7 sessions (median = 7) out of 8 sessions total, and facilitator fidelity was very good. Of the women who completed class evaluations, at least half reported that each of the three class components (mindful breathing, mindful eating, and mindful movement) were “very useful,” and that they used them on most days at least once a day or more. Women improved in reported levels of mindfulness, acceptance, and emotion regulation, and these increases were correlated with reductions in stress, depression, and overeating.

**Conclusions:**

These findings suggest that in pregnant women at high risk for excessive weight gain, it is both feasible and effective to use mindfulness strategies taught in a group format. Further, increases in certain mindfulness skills may help with better management of stress and overeating during pregnancy.

**Trial registration:**

ClinicalTrials.gov NCT01307683, March 8, 2011.

**Electronic supplementary material:**

The online version of this article (10.1186/s12884-018-1757-6) contains supplementary material, which is available to authorized users.

## Background

Obesity is a leading public health concern in the United States [1]. In 2013–2014, the prevalence of overall obesity (body mass index [BMI] ≥30) was 40.4% among adult women, and 9.9% for class 3 obesity (BMI ≥40), with a significant linear increase over time between 2005 and 2013–2014 [2]. The prevalence of obesity for black (57.2%) and Latina/Hispanic (46.9%) women far exceeds that of white women (38.7%). Women who are already overweight or obese before pregnancy have greater risk for gestational weight gain greater than the recommended 15–25 pounds, or 11–20 pounds, respectively [3].

Both pre-pregnancy obesity and excessive maternal weight gain during pregnancy are associated with a number of unfavorable outcomes. Obesity during pregnancy increases risks of antenatal obstetric problems, caesarean delivery, and fetal risks [4] and gestational weight gain in excess of IOM guidelines has been linked to neonatal complications [5, 6]. Excessive weight gain during pregnancy is also directly associated with increased BMI and risk of obesity in offspring into adolescence [7]. Current studies suggest that obese women gaining no or low gestational weight have better health outcomes [8]. Pregnancy may be a unique opportunity to positively influence a woman’s weight gain trajectory by offering low-cost, feasible, and effective interventions that could reduce health risks for both mother and child.

Obesity and weight gain cannot be remedied through a simplistic approach to reducing caloric intake. Food, medications, physical inactivity, toxins, and viruses interact with genetics to interfere with energy balance and contribute to obesity [9]. Weight gain is fostered in some by non-homeostatic eating (defined as eating reflexively in response to factors other than caloric need or hunger), and includes mindless eating, reward-based eating, and stress eating [10–12], which are especially common during periods of chronic stress [13–15]. The drive to eat non-homeostatically is primed by strong neurobiological signals involving reward and stress systems [10]. For example, palatability of high fat and sweet foods is heightened with stress [16] by increasing cortisol, which in turn stimulates dopamine activity. Secretion of stress-related glucocorticoids also increases motivation for food and secretion of insulin, which promotes food intake and obesity [17]. This pattern is reflects what has been observed in animal studies on the neurobiology of addiction and drug abuse [18, 19].

Maternal obesity during pregnancy confers increased risk of her child becoming overweight through a number of biopsychosocial mechanisms such as maternal and fetal hyperglycemia and hyperinsulinemia [20], stress-induced excessive gestational weight gain [21, 22], and stress-induced epigenetic changes leading to offspring fat storage and obesity [23].

Current dietary approaches to healthy weight gain during pregnancy focus almost exclusively on nutrition education and individual control of food intake [24]. Programs based solely on nutritional recommendations have limited success in preventing excessive gestational weight gain [25, 26], perhaps because they rarely address the root causes of the strong drive to overeat. A successful program to address excessive weight gain during pregnancy must also integrate behavioral strategies for managing appetitive drive, knowledge of the psychology of eating and behavior change, and the neuroscience of stress, appetite, and reward.

Mindfulness-based interventions are receiving increasing attention and empirical support for reducing stress and addressing stress-related medical problems and behaviors [27–29]. Mindfulness skills may be important for combating non-homeostatic eating in three ways. First, stress is a fundamental but sometimes overlooked factor that both predicts and interacts with eating behaviors to increase risk for obesity. Mindfulness skills can target the stress-eating interaction because they are effective for reducing anxiety, depression, and stress [30, 31], which should in turn help to reduce stress-eating. Second, the psychological causes of emotional eating are believed to involve poor awareness of internal physiological states and differentiation of hunger cues and emotional arousal [32, 33]. Mindfulness increases attention to and awareness of internal sensations, which may help individuals develop a heightened ability to differentiate hunger cues from emotion responses, and detect and respond to satiety cues. Consequently, the frequency of binge eating can be reduced [34–37]. Third, mindfulness practices are aimed at increasing attention to and awareness of thoughts and emotions. Emotional eating is thought to be a self-regulation process, where one’s attention is shifted away from negative affect or negative self-appraisals towards an immediately available reward-stimulus such as food [38, 39]. Mindfulness allows individuals to gain awareness of, acknowledge, and tolerate their internal states without immediately responding to them, thus possibly reducing their need to shift their attention to food. At the same time, these practices may facilitate awareness of the thoughts and feelings that trigger emotional eating. In addition, mindfulness-based interventions are short-term, low-cost, and feasible to integrate into standard prenatal care [40–42].

**Fig. 1:**
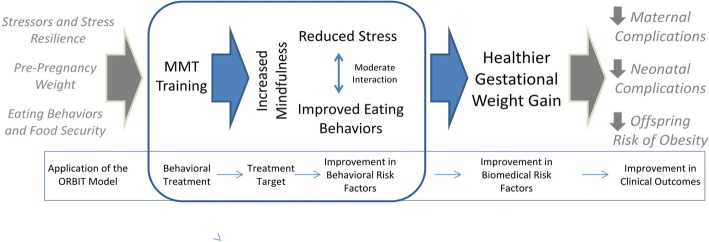
Theoretical Model

The aim of this project was to develop a psychoeducational intervention for overweight or obese pregnant women to encourage better nutrition and healthy weight gain during pregnancy by teaching mindfulness skills for stress reduction integrated with nutritional and exercise recommendations. The overall project utilized the Obesity-Related Behavioral Intervention Trials (ORBIT) model for developing behavioral treatments to prevent and/or manage chronic disease [43]. Our specific model (see Fig. 1) was that women enter into pregnancy with a diverse array of factors influencing pre-pregnancy weight and nutrition, including life stressors and stress-resilience, food security/availability, and eating behavior patterns. We hypothesized that the Mindful Moms Training (MMT) would, through increasing mindfulness of stress, hunger, fullness, and satiety cues, reduce stress and increase resilience to stressors, improve eating behaviors, and modify to a certain extent the coupling between stress and overeating. By doing this, we hoped that the intervention would result in healthy gestational weight gain within IOM guidelines, and theoretically improve maternal outcomes, neonatal outcomes, and offspring outcomes postpartum. The area within the curved blue box illustrates the portion of the study this paper focuses on: development of the intervention and assessment of its impact on hypothesized mediators. The rectangular box indicates how our model follows the application of the ORBIT model.

In this paper we describe a) our process model for intervention development; b) the content of the intervention resulting from this process; c) attendance, feasibility and teacher fidelity to the intervention; d) the intervention’s effects on proposed mechanisms of change (mindfulness, acceptance, emotion regulation); and e) whether these were related to well-being (reductions in stress, depressed mood) and non-homeostatic eating behaviors (emotional eating and external eating), focusing only on the participants who received the intervention (not including the comparison group).

## Methods

The Maternal Adiposity, Metabolism, And Stress (MAMAS) study was funded by a National Institutes of Health U01 collaborative research mechanism intended to facilitate developmental approaches to translating basic research into effective behavioral interventions for chronic disease. We used the Obesity-Related Behavioral Intervention Trials (ORBIT) model of intervention development [43], which outlines clear phases for a flexible and progressive process, with clinically relevant milestones for forward movement and return to earlier stages for refinement and optimization.

The MAMAS study began with focus groups with overweight and obese pregnant women (*n* = 59) representative of the target population to assess interest in, and identify potential barriers to, participating in a mindfulness-based prenatal behavioral intervention for health weight gain during pregnancy [44, 45]. We found that the participants in our target population faced substantial stressors in multiple domains of financial, relationship, pregnancy-related, and weight and health-related situations. They were very interested in the idea of a stress-reduction intervention and were open to the mindfulness approach that we described. Next we conducted a proof-of-concept pilot study, which informed recruitment methods, data-driven selection of intervention content, and intervention delivery logistics. This was followed by the MAMAS intervention trial, which is the primary focus of this paper. We first describe the overview of the study and sample, then intervention development and content, and end with quantitative analyses of proposed mechanisms.

### Participants and setting

Participants in the San Francisco Bay Area were recruited between 8 and 20 weeks gestation and screened for eligibility, completed clinical assessments, and began the intervention between 12 and 20 weeks gestation. Inclusion criteria were: being pregnant, age 18 to 45 years, pre-pregnancy BMI minimum = 25 and maximum = 41 or < 300 pounds, and income to poverty ratio ≤ 500% (median income to poverty ratio in the Bay Area, specific to family size). We utilized a wide array of direct and indirect recruitment strategies and found that in-person recruitment at hospital-based prenatal clinics produced the highest yield of participants. Establishing close relationships with prenatal care providers, clinic staff, social service agencies and study participants was an essential precursor to successful recruitment and retention of our study participants [45].

Prospective participants engaged in a group or individual orientation session to learn more about the study requirements and informed consent was obtained from those who chose to participate [46]. A total of 110 pregnant women were recruited to participate in 11 waves of the intervention with 7–13 participants in each wave between 2011 and 2013. One hundred non-randomized treatment-as-usual participants were recruited as a comparison group: their data are not provided in this paper. Orientations and intervention sessions were conducted at CPMC’s St. Luke’s Hospital and community health centers in the San Francisco Bay Area. The clinical assessments took place at the UCSF General Clinical Research Center where hospital emergency care was immediately available.

### Intervention development

Intervention development proceeded in three stages. First, we created an initial provisional intervention. Second, the intervention was refined and optimized through an iterative process over the course of eleven waves of participants (*n* = 110). Participants in each wave received successive versions of the intervention. Intervention facilitators were selected based on three criteria: 1) experience facilitating mindfulness-based health interventions, 2) experience working with pregnant women and new mothers matching the target population, and 3) willingness to adhere to a research protocol by a) adhering to a manualized intervention, b) providing facilitator feedback, and c) participating in intervention refinement. Two facilitators were selected for this project, one a Masters-level psychology student, and the other a certified nurse midwife (CNM) and family nurse practitioner (FNP), both with extensive experience working with diverse populations formally training in mindfulness-based interventions. Both were trained and supervised by the primary MMT intervention developers (CV and JK). Third, we assessed participants’ retention, engagement and subjective response to the intervention and examined pre- to post-intervention changes in hypothesized mediators of mindfulness, mindful eating, activity levels, and acceptance-based coping.

#### First stage of intervention manual

The initial intervention manual was created using a problem-formulation approach [47] which calls for tailoring interventions to match the population and problem being addressed. We developed the initial content and structure of the intervention based on a theory of change, literature review, and qualitative analysis of focus group responses of 59 stressed, low-income, overweight/obese pregnant women [44]. We then selected components from existing mindfulness-based eating and stress reduction interventions that have shown promise for improving eating, stress, and mood [40, 48–52] and would be suitable to the specific sources of stress, needs and capacities of the target population. These data also informed recruitment materials, reading levels and language used to describe concepts, and incentives for participating.

We selected intervention components designed to achieve the intended outcomes (healthy gestational weight gain and reduced distress) through our theorized mechanisms of action (eating behavior, mindfulness of food choices and hunger, fullness, and taste satiety cues; acceptance-based coping, mindfulness, and improved emotion regulation through reappraisal rather than suppression of distress). Components were selected that demonstrated evidence for 1) reducing distress and improving mood in pregnant women and new mothers (the Mindful Motherhood Training [53]); 2) reducing overeating and binge eating (Mindfulness-Based Eating Awareness Training (MB-EAT) [54]) and Supporting Health by Integrating Nutrition and Exercise (SHINE) [49]). These components were modified to be appropriate for the target population, and to more directly target the stress-overeating interaction. The overall format from these mindfulness intervention derivatives were inspired by Mindfulness-Based Stress Reduction (MBSR) [55]. Like other mindfulness-based interventions that have been empirically supported, an eight-week length of intervention was determined to be suitable for delivering information in a relatively brief window that would provide enough time for participants to introduce and incorporate new concepts and behaviors, while allowing for potential short-term changes in weight gain trajectories.

#### Intervention manual refinement

Intervention sessions were audio-recorded and the intervention facilitators met weekly with clinical supervisors (CV and JK) by phone to review and discuss feasibility, effectiveness, and areas for improvement. Changes to the initial intervention were made through an iterative process based on: 1) weekly facilitator feedback; 2) supervisor review and assessment of audio recordings of sessions, and 3) mid-course and post-course participant evaluations. Proposed changes were made in consensus decision making sessions among the curriculum development team and were then included in the manual for the subsequent wave. Substantial changes were made during waves 1–5, such as simplifying the movement series and moving it to the beginning of each session, reducing time spent on and simplifying delivery of stress-reduction concepts, increasing focus and time spent on nutritional recommendations with more concrete examples and the “what to eat, how to eat, and how much to eat” framework, and changing exercises and metaphors to ones that were more relatable to the population (such as stress from feeling overwhelmed at work to stress from having the car break down on the freeway). More subtle refinements were made during waves 6–11, such as creating more time for question and answer periods after specific exercises, and making exercises more interactive to assure participants were engaged and following the material. The process led to several key decisions about the structure and content of the training, and changes from the provisional version to the resulting final intervention.

#### Final intervention

The resulting intervention, the Mindful Moms Training (MMT), is an experiential training where pregnant participants attend a two-hour group session once a week for eight weeks and are asked to engage in assigned readings and experiential practice daily between sessions (note: the terms “training” and “session” are used intentionally rather than “class” to imply an active rather than passive stance of participants). Each session begins with a period of mindful movement, such as gentle stretching and beginner level yoga (~ 15 min), followed by a verbal check-in regarding home practice and progress toward goals in the previous week (~ 15 min), discussion of a mindful stress reduction topic (~ 15 min), mindful eating and nutritional recommendations (~ 30–40 min), mindfulness practice (~ 15 min), review of home practice and goals for the coming week (~ 10 min), and review/checkout/closing (~ 10 min).

Participants were asked to complete all 8 sessions to the best of their ability, and participants who thought they might not be able to attend more than two sessions at the scheduled times were not enrolled. Participants who missed a session were called the next day by the teaching assistant, and the content of the session and recommended homework was reviewed with them. Class sessions were held at 5:30 pm on a weekday for the most part, which was determined through focus groups to be the best time for most pregnant women since many of them worked during the day, or needed to wait until a partner or grandparent came home from work to care for other children. Participants were welcomed to bring food to the sessions since they occurred during most people’s dinner time. We provided incentives for attending group sessions including free gifts such as water bottles, yoga mats, baby clothing, and other items donated by local companies. Each participant was reimbursed $25 at the end of each session for childcare and transportation costs. We did not offer childcare due to potential the liability involved.

The training focuses equally on 1) nutritional and eating behavior recommendations, 2) mindful awareness of hunger, fullness, taste satiety, and food choices, and 3) mindfulness skills for stress reduction (sitting meditation, gentle stretching, acceptance-based stress coping concepts, and informal practices in daily living) (Table 1). A strong social support component organically emerged in which women described feeling relief through sharing their experiences and hearing about the experiences of other women.

1. *Nutrition and Eating Behavior Recommendations.* The nutritional components included 1) “What to Eat” - including recommendations for choosing more fresh whole foods, and less high sugar/processed foods, discussion of nutritional content, recipes and cooking instructions, drinking more water, replacing foods with more nutritional options, and reading or scanning labels & calories; 2) “How Much to Eat” - discussion of portion sizes and proportions of vegetables, proteins, grains, fruits, dairy, and fats, using the plate method and food pyramid as tools, and 3) “How and When to Eat” - discussion of changing unhealthy eating behaviors such as eating in front of the TV or eating chips out of the bag, and encouraging healthy eating behaviors like eating more small meals per day rather than few large ones, and using small-sized plates.
2. *Mindful eating.* The mindful eating portions of the course were adapted from the Mindfulness-Based Eating Awareness Training (MB-EAT) program [56, 57]. MB-EAT focuses on fostering mindful awareness while eating (i.e., paying attention rather than distracting or “zoning out” while eating). In particular, participants learned and practiced in class: 1) mindful awareness of hunger and fullness; 2) mindful awareness of taste satisfaction and satiety; and 3) mindful awareness of food choices. They also learned to be more aware of thoughts and feelings related to eating, including discussion of stress and emotional eating, and learning how to use mini-meditations before meals. A key element of this component of the program were in-depth experiential eating exercises using food in class, such as exploring taste satiety through mindful eating of potato chips, learning to rate level of hunger prior to eating a piece of chocolate cake, and assessing fullness while drinking a bottle of water. As in MB-EAT, we made the distinction that mindful eating entailed cultivating “inner wisdom,” while adapting nutritional recommendations to personal needs was “outer wisdom,” so that participants could understand the distinction between the two and utilize both in their eating behaviors and food choices.
3. *Mindfulness for stress reduction.* Content for this portion was adapted from Mindfulness-Based Stress Reduction [58], Mindfulness-Based Cognitive Therapy [52], and the Mindful Motherhood Training [40], with inspiration from Mindfulness-Based Childbirth and Parenting [41]. Elements of Mindful Self-Compassion training [59] were included as well, since many women reported experiencing stress from their own perceived failings. This was also designed to buffer against stress potentially caused by the intervention’s focus on weight and eating, which for many women can result in shame or self-criticism. The stress reduction components of the course were delivered through interactive discussion, small group and dyad work, and experiential exercises focused on acceptance-based coping techniques.

**Table 1:** Mindful Moms Training primary intervention components

Nutritional/Eating Behavior	Mindful Eating	Mindful Stress Reduction
What to Eat	Mindful vs. Mindless Eating	Acceptance-Based Coping
How Much to Eat	Hunger and Fullness	Present Moment Awareness
How and When to Eat	Taste Satisfaction and Satiety	Awareness of Breathing
	Food Choices	Body Scan
	Stress and Emotional Eating	Observing Thoughts and Feelings
	Mini-Meditations Before Meals	Mindful Connection with Baby
		Self-Compassion

Five types of mindfulness were taught: awareness of breathing, awareness of body sensations (both sitting and while moving), awareness of thoughts and feelings, awareness of connection with the baby, and mindful awareness in everyday life (including eating, but also extending to such activities as being mindful while showering, or while washing dishes). It also included mini-discussions on topics such as “The Observing Self,” “What is Acceptance?,” “Thoughts are not Facts,” “Focusing on the Present Moment,” “Mindfulness in Relationships,” “Mindful Decision Making,” and “Self-Compassion.” We found it very important to make these topic discussions highly interactive rather than didactic, using Socratic methods, metaphors, and frequent real-world examples.

**Fig. 2:**
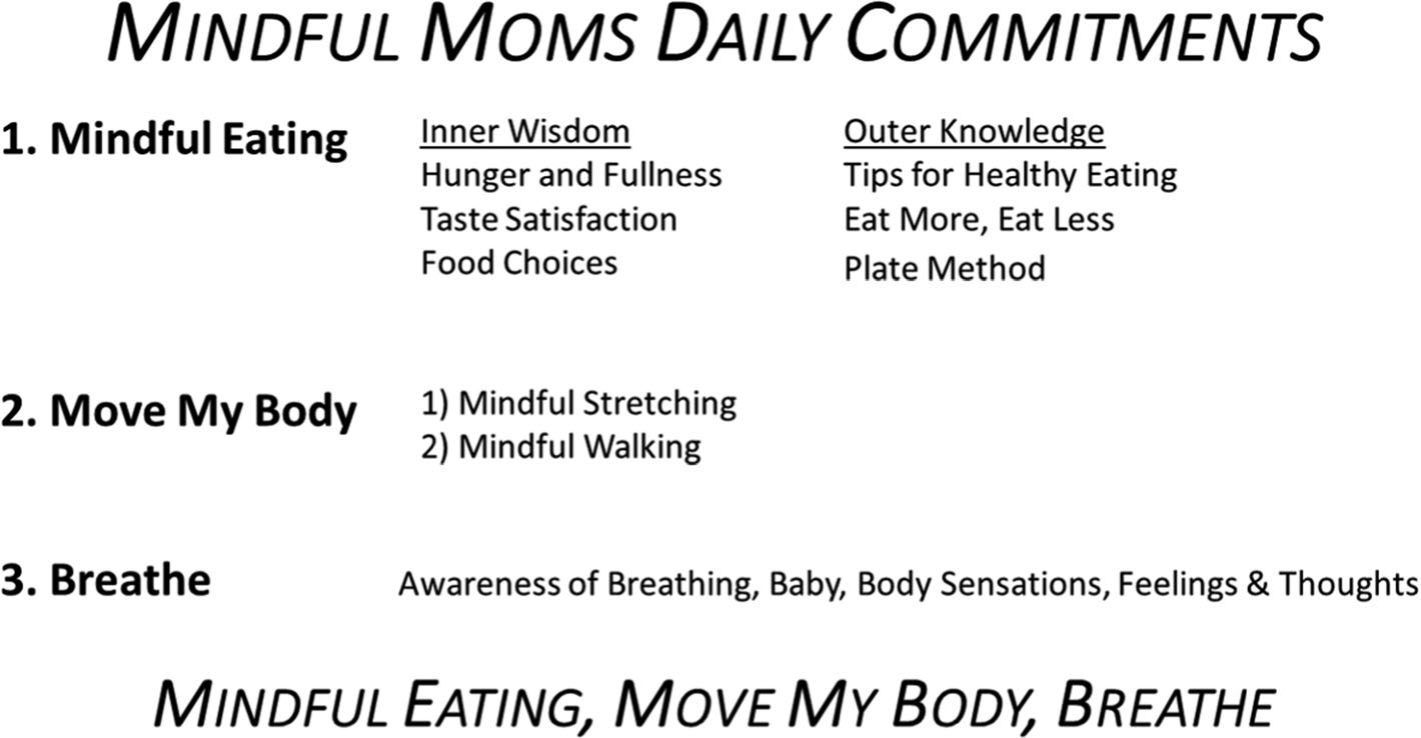
Mindful Moms Three Daily Commitments

The overall course content was summarized for participants in what we called the “Three Commitments” represented by the slogan “Mindful Eating, Move My Body, Breathe!” (see Fig. 2). Throughout each session, the three commitments were reinforced. Participants were provided with laminated cards summarizing the primary program components (see Fig. 1). At the beginning of each session, the group discussed progress and obstacles from the previous week and troubleshooting, problem solving, and goal setting for the coming week. At the end of each session, homework for the coming week was reviewed (e.g., reading assignments, mindful eating, nutrition and exercise recommendations, and recorded guidance for mindful awareness practice). A brief practice of compassion/self-compassion meditation closed each session.

### Measures

An array of measures was administered as part of the larger MAMAS study and are reported elsewhere (Epel E, Laraia B, Coleman-Phox K, Leung C, Vieten C, Mellin L, Kristeller J, Thomas M, Stotland N, Bush N et al: Effects of a mindfulness-based intervention on distress, weight gain, and glucose control in pregnant low-income women: a controlled trial, in preparation, [60]) (see Additional file 1 – MAMAS Study Demographics Questionnaire in supplementary materials online). Measures specific to the intervention development portion of the project are reported here:

1. *Participant Retention/Attendance.* Participants were asked to report how many hours they practiced homework per week, and attendance at sessions was tracked by facilitators.
2. *Instructor Fidelity Assessment*. All session were audio-recorded, and between 2 and 5 sessions from each wave were randomly selected to be assessed by trained graduate psychology students, using a checklist for the extent to which facilitators delivered the primary components of the intervention as outlined in the manual (see Additional file 2 – MAMAS Study Fidelity Assessments in the supplementary materials online). A total of 32 sessions were reviewed.
3. *Participant Response.* A Final Evaluation Questionnaire was given to participants in Waves 1–11 at the end of the final class for each wave or was mailed to participants who did not attend the last class (see Additional file 3 – MAMAS Study Final Evaluation in supplementary materials online).
  Fifty-eight questionnaires were returned, of 110 total participants. The questionnaire included structured and open-ended questions about the program including convenience of class location and time, reasons for enrolling, teacher attention, satisfaction with the program, likes and dislikes, usefulness and frequency of use of program components (Mindful Awareness of Breathing, Mindful Movement, Mindful Eating), comprehension of mindfulness and acceptance, use of mindfulness skills outside of program, and amount of time outside of program spent using new skills each week.
4. *Self-Report Questionnaires.* Participants were asked to complete baseline measures upon enrollment and post-intervention measures nine weeks later, including:
  The *Five Facet Mindfulness Questionnaire (FFMQ)* [61] assesses the general tendency to be mindful in daily life with subscales to assess respondents’ ability to act with awareness (α = .86), observe experiences (α = .78), describe feelings (α = .91), non-judging (α = .86), and non-reactivity (α = .73) [62]. The FFMQ has been shown to have convergent and discriminant validity in relation to other psychological constructs in meditating and non-meditating samples. The intervention was designed to increase mindfulness generally, and thus all subscales were included.

To assess the potential mechanism of mindful eating, we utilized two scales from the *Mindful Eating Questionnaire (MEQ)* [63]: mindful awareness of eating, such as noticing flavors, sweetness, colors, and smells of food) (α = .74), and eating in response to external cues (e.g. eating because the food is there, such as eating popcorn in a movie theater or candy from a dish) instead of in response to hunger, (α = .70).

The *Acceptance and Action Questionnaire (AAQ)* [64] is a ten-item questionnaire that measures psychological flexibility, or the ability tolerate negative thoughts and feelings (acceptance) in the pursuit of goals or, depending upon the situation, change behavior to accomplish goals (action) (α = .84). The intervention was designed to increase psychological flexibility/acceptance and decrease experiential avoidance.

The *Emotion Regulation Questionnaire (ERQ)* [65] is a 10-item questionnaire that assesses positive reappraisal (the ability to reframe or look at situations in a positive light) (α = .79), and emotional suppression (the tendency to suppress and avoid negative emotional responses) (α = .73).

The *Perceived Stress Scale (PSS)* [66] was used to measure the degree to which situations in one’s life are appraised as stressful, or how unpredictable, uncontrollable, and overloaded respondents find their lives (α = .91). The *Patient Health Questionnaire (PHQ-9)* [67] was used to measure depressive symptoms (α = .86–.89) [68].

To assess eating behavior outcomes, we utilized two scales of the *Dutch Eating Behavior Questionnaire* [69]: emotional eating (α = .93), or overeating in response to emotions, and external eating (α = .80), or eating in response to food-related stimuli, regardless of the internal states of hunger and satiety.

### Statistical analyses

We compiled descriptive results (means, standard deviations, or frequencies) for teacher fidelity to the critical domains of the intervention, class attendance and adherence. To assess change in presumed mechanisms, we used paired *t*-tests comparing baseline and endpoint scores. Pearson correlation coefficients were calculated to evaluate changes in general mindfulness, acceptance, mindful eating correlated with perceived stress score changes. All analyses were conducted using SAS software [70].

## Results

### Demographics

As shown in Table 2, participants in the intervention were an average of 28 years old, predominantly low income, women of color, 78.2% had a high school diploma and/or some college or vocational training, and 13% had a college degree. Thirty-three percent were single, separated, or divorced. There was a notable amount of food insecurity (42%) [71]. Around 45% were obese, and 55% were overweight. Women started the intervention at a mean of 15.9 (SD = 3.8) weeks gestation and completed it at a mean of 24 (SD = 3.0) weeks gestation.

**Table 2:** Demographics

	MMT (*n* = 110)
Age (years), mean (SD)	27.8 (5.7)
	N (%)
Race/ethnicity	
White	14 (12.8)
African American	39 (35.8)
Latino	35 (32.1)
Other/ Multiracial	21 (19.2)
Education	
< 12 years	10 (9.1)
High school graduate/GED	30 (27.3)
Any college or vocational training	56 (50.9)
College graduate or higher	14 (12.7)
Marital status	
Married or in committed relationship	74 (67.3)
Single, separated or divorced	36 (32.7)
Household income, mean (SD)	$24,723 ($22,459)
Number of previous children, mean (SD)	0.8 (1.0)
Pre-pregnancy weight status	
Normal or overweight	58 (55.2)
Class I obese	30 (28.6)
Class II obese	17 (16.2)
Food-Insecure	44 (41.9)
Smoking status	
Current smoker	5 (4.8)
Former smoker	44 (42.3)
Never smoker	55 (52.9)
Leisure-time physical activity	
Inactive or light activity	58 (56.9)
Moderate or vigorously around 3 times/week	21 (20.6)
Moderate or vigorously on most days	23 (22.6)

### Participant retention/attendance

Of the 114 women enrolled in the intervention, eight women did not complete the baseline questionnaires and thus their quantitative data are not reported here. Four women miscarried and 7 were lost to follow up (1 moved, 1 unable to attend classes, and 5 unable to contact). Participants attended a mean of 5.73 (*SD* 2.31) sessions out of 8 with a median of 7 (see Table 3 for number of participants and percent of total sessions attended by wave). Overall, the mean number of classes attended was 0.79 (SD .06) On average, women reported spending 4.1 h (range 0.16–17.5) outside of class practicing the new skills they learned each week.

**Table 3:** Participant percentage of total sessions attended by wave

Wave	% of total sessions attended	SD	N
W1	0.83	0.29	8
W2	0.71	0.26	12
W3	0.84	0.23	12
W4	0.84	0.32	7
W5	0.69	0.28	10
W6	0.78	0.23	8
W7	0.83	0.15	9
W8	0.88	0.19	6
W9	0.75	0.26	13
W10	0.84	0.28	9
W11	0.75	0.36	12
Total	0.79	0.06	106

### Facilitator Fidelity

Intervention fidelity was good. In 93% of the sessions reviewed, the intervention facilitators “mostly” or “completely” implemented the “Move My Body” gentle stretching aspect of the intervention. In 88% of the sessions the facilitators “mostly” or “completely” provided the “Mindful Eating” portion of the intervention. In 69.2% of the sessions facilitators “mostly” or “completely” delivered the “Breathe” mindfulness-stress reduction component. Facilitators “completely” reviewed the three commitments at the end of each session 88.5% of the time, and 73% of sessions ended in the compassion meditation as indicated in the manual.

### Participant response

Fifty-eight women (53%) completed a Final Evaluation Questionnaire. The lower response was due to the questionnaire being sent after the completion of the post-intervention research assessment. We strongly recommend in future studies that program evaluation questionnaires be completed in class or at assessment visits to improve response rates. Among those who responded, most reported that the class location was either “very convenient” (60%) or “somewhat convenient” (29%), as was the day and time of the class (“very convenient” 55%; “somewhat convenient” 41%). Almost all participants felt they had enough opportunities to ask any questions they had of the teachers during or after class (95%; 3% did not have any questions to ask). Most participants were “very satisfied” 71% or “satisfied” 29% overall. No one endorsed being “dissatisfied” or “very dissatisfied”. All participants reported understanding of the idea of acceptance (“understood it very well” 65%; “understood it somewhat” 35%).

Participants reported finding the three major program components useful, and most participants practiced at least one mindfulness skill at least a few times over the past week (Table 4). The eating and movement advice for outside of class (the three basic commitments and tips for eating healthy and movement) was also found to be useful (“very useful” 46%; “useful” 49%; “not very useful” 4%; and “not at all useful” 2%). Ninety-five percent of the participants used one or more of the skills or concepts learned in the program outside of class. Mindful breathing was most endorsed (48%), followed by mindful eating (36%), and mindful movement (33%).

**Table 4:** Participant reported usefulness and frequency of use of intervention components (*n* = 58)

	How useful?				How often used over past week?			
Program Components	Not at all useful	Not very useful	Useful	Very useful	Did not use	A few times	1×/day most days	Several times most days
Mindful Awareness of Breathing	0%	3%	41%	55%	2%	31%	40%	27%
Mindful Movement	0%	7%	44%	53%	7%	32%	30%	32%
Mindful Eating	0%	7%	40%	53%	11%	39%	30%	20%
Three Basic Commitments	2%	4%	49%	46%	N/A	N/A	N/A	N/A

### Changes in aspects of mindfulness and emotion regulation

We assessed changes in mindfulness in several domains (mindful eating, psychological flexibility/acceptance, the five facets of mindfulness captured by the FFMQ, and emotion regulation), from pre-intervention to post-intervention, within subjects. There were significant increases in mindful eating (awareness of eating, *p* < .01), and non-significant reductions in external eating (*p* = .09). There were improvements in three of the five mindfulness subscales of the FFMQ: observe (*p* < .0001), non-judging (*p* = .03), and non-reactivity (*p* = .002) with a non-significant trend toward improvement in the “act with awareness” subscale (*p* = .08). There were also increases in AAQ psychological flexibility/acceptance (which can also be described as reductions in experiential avoidance) (*p* < .0001), and in adaptive emotion regulation in the tendency toward reappraisal (*p* = .002), with no change in tendency for emotional suppression (see Table 5). There were significant improvements in PSS perceived stress (*p* < .0001) and PHQ depression (*p* < .0001) from pre-intervention to post-intervention.

**Table 5:** Changes in mindfulness and emotion regulation from pre- to post- intervention

	Pre	Post	Change in Intervention Group^a^
	Mean (SD)	Mean (SD)	P
Mindful Eating – Awareness (MEQ) *N* = 79	2.55 (0.6)	2.73 (0.7)	**0.01**
Mindful Eating – External (MEQ) *N* = 77	2.31 (0.6)	2.43 (0.8)	0.09
Psychological Flexibility (AAQ) *N* = 80	49.92 (10.1)	53.67 (8.9)	**< 0.0001**
Mindfulness – Observe (FFMQ) *N* = 81	25.64 (6.2)	28.61 (6.1)	**< 0.0001**
Mindfulness - Describe (FFMQ) *N* = 81	29.05 (5.6)	29.67 (5.2)	0.19
Mindfulness - Act w/Awareness (FFMQ) *N* = 80	28.40 (6.0)	29.28 (5.0)	0.08
Mindfulness - Nonjudge (FFMQ) *N* = 80	27.48 (5.9)	28.76 (5.6)	**0.03**
Mindfulness - Nonreact (FFMQ) *N* = 80	19.60 (4.7)	21.27 (4.0)	**0.002**
Emotion Regulation - Reappraisal (ERQ) *N* = 78	28.29 (6.5)	30.46 (6.5)	**0.002**
Emotion Regulation - Suppression (ERQ) *N* = 80	12.51 (5.0)	12.88 (4.8)	0.51
Perceived Stress (PSS) *N* = 82	18.62 (6.1)	15.77 (5.7)	**< 0.0001**
Depression (PHQ9) *N* = 82	7.12 (5.6)	4.57 (3.8)	**< 0.0001**

^a^Paired *t*-tests

### Correlations between changes in targets and outcomes

As shown in Table 6, as hypothesized, there was a pattern showing that increases in measures of mindfulness were associated with decreases in distress (stress and depression) and self-reported eating behavior. Specifically, increases in psychological flexibility (acceptance) were significantly correlated with decreases in stress, depression and emotional eating (but not external eating). Similarly, improvements in all five of the mindfulness (FFMQ) subscales were correlated with decreases in either stress or depression or both. Increases in the mindfulness subscales measuring “acting with awareness” and “non-judging” were correlated with a decrease in emotional eating and external eating. Finally, increases in the reappraisal form of emotion regulation were correlated with decreased stress and emotional eating, whereas increases in emotion suppression were correlated with increased stress, depression, and a non-significant increase in emotional eating. There were no associations between the mindful eating subscales of “awareness” and “external eating” with stress, depression, or eating behaviors.

**Table 6:** Pearson correlations between change in mindfulness measures and changes in distress and eating behavior

Changes in Mindfulness Variables	n	Change in Perceived Stress (PSS)	Change in Depression (PHQ-9)	Change in Emotional Eating (DEBQ)	Change in External eating (DEBQ)
Mindful Eating Questionnaire					
Mindful Eating - Awareness (MEQ)	58–79	−0.10	−0.02	−0.18	−0.02
Mindful Eating - External (MEQ)	56–77	−0.00	−0.08	−0.06	0.04
Acceptance and Action Questionnaire					
Psychological Flexibility (AAQ)	59–80	− 0.26*	− 0.40***	− 0.22*	− 0.02
Five Factor Mindfulness Questionnaire					
Mindfulness - Observe (FFMQ)	59–81	− 0.33**	− 0.06	− 0.17	0.00
Mindfulness - Describe (FFMQ)	59–81	− 0.36***	− 0.42***	− 0.13	− 0.12
Mindfulness - Act With Awareness (FFMQ)	59–80	− 0.18	− 0.46***	−0.25*	− 0.32**
Mindfulness - Nonjudge (FFMQ)	59–80	−0.14	− 0.26*	−0.28*	− 0.33**
Mindfulness - NonReact (FFMQ)	58–80	−0.36**	− 0.13	−0.16	− 0.05
Emotion Regulation Questionnaire					
Emotion Regulation - Reappraisal (ERQ)	58–78	−0.28*	−0.03	− 0.25*	−0.19+
Emotion Regulation - Suppression (ERQ)	59–80	0.22*	0.26*	0.18	0.13

*** = *p* < .001, ** = *p* < .01, * = *p* < .05, + = *p* < .10

The sample size (n) varies, ranging from 56 to 80, as shown, due to missing data

## Discussion

In this study, we developed and tested an eight-week mindfulness-based intervention directed toward reducing stress and overeating in pregnancy. We have described both the content of the intervention, and the iterative process of intervention development required to adequately tailor and optimize the intervention for a high-risk sample. We utilized the ORBIT model, which recognizes that interventions must be customized to meet the needs of special vulnerable populations, and intervention development must be strongly informed by iterative feasibility testing with the target population.

The resulting Mindful Moms Training (MMT) is a mindfulness-based psychosocial intervention for low- to middle-income overweight/obese pregnant women that was designed to foster healthy weight gain during pregnancy and reduction of transmission of obesity to infants by targeting 1) reductions in stress and negative mood, through acceptance-based coping, and 2) improved nutrition and healthy eating behavior during pregnancy through mindful eating practices, stress reduction, and increased activity.

We were able to obtain excellent retention, attendance, and reporting of home practice outside of the class setting. Attendance was similar across waves. Participants reported high satisfaction with the program, in terms of content and logistics. We also found strong facilitator fidelity to the intervention.

Partial support was found for the hypothesized mechanisms of change: general mindfulness, mindful eating, acceptance, and emotion regulation. We found improvement in awareness of eating, and three facets of mindfulness - the ability to observe inner experiences (e.g., distressing thoughts, sensations, or emotions), nonjudgment, and non-reactivity to those experiences. In addition, we found increases in psychological flexibility, which is defined by greater acceptance of experiences (i.e., reductions in experiential avoidance), as well as the ability to regulate emotions by reappraising situations. Further, we found evidence that improvements in mindfulness were correlated with decreases in stress, depression, and emotional and external eating (i.e., eating to soothe distress or in response to environmental cues instead of hunger or caloric need).

These results support the presumed mechanisms of mindfulness practices in reducing distress and improving eating behavior in pregnant women. The primary outcomes from the nonrandomized trial that utilized this intervention (reported elsewhere (Epel E, Laraia B, Coleman-Phox K, Leung C, Vieten C, Mellin L, Kristeller J, Thomas M, Stotland N, Bush N et al: Effects of a mindfulness-based intervention on distress, weight gain, and glucose control in pregnant low-income women: a controlled trial, in preparation)) showed that women who were enrolled in MMT, compared to a non-randomized comparison group of women receiving treatment as usual, had significant reductions in perceived stress, depressive symptoms, and glucose levels after an oral glucose tolerance test (glucose regulation), but no differences in whether they met Institute of Medicine (IOM) criteria for recommended weight gain. Although there were no detectable effects on the primary outcome of weight, the positive effects on mothers’ mental health are important. Those in MMT had lower depression, not just post-intervention but throughout the postpartum period [72]. Additionally, their offspring had fewer medical visits [73]. We are currently following this sample to examine developmental effects on offspring.

Our results should be interpreted while bearing in mind the study’s limitations. We had a substantial rate of missing final participant evaluations of the intervention that may have led to biased results, and could not compare those who completed the final evaluations with those who did not because we collected them anonymously (to help participants feel completely free to be critical in light of the power differential). Also, because the intervention was developed through an iterative process over the course of the study, each wave of participants provided data based on slightly different intervention designs. While statistically significant, changes in mindful eating (awareness) and mindfulness (observe, nonjudge, and nonreact) subscales were small (< 12%). Since there are not yet norms established for clinically meaningful improvements, it is possible that these improvements are clinically negligible. It is also possible that even minor improvements in these variables can make a difference in the outcomes of interest. Reductions in perceived stress and depression were 15 and 36% respectively, which are more noticeable clinically. Reported relationships between increased mindfulness/acceptance and reduced stress, depression, emotional eating and external eating were based on correlations, and thus, we cannot infer causal relationships. Power was not adequate to conduct a more thorough analysis of the relationships between these change scores while controlling for potentially confounding variables.

Another limitation of this project was that pregnant women began the intervention in the second trimester, which is potentially late for addressing eating behaviors and weight gain. It is difficult to recruit subjects and implement a group intervention during the first trimester since many women do not realize that they are pregnant, or trust in the viability of the pregnancy, until the end of the first trimester. Future studies could focus on pre-pregnancy women, or provide a drop-in style group format immediately upon confirmation of pregnancy.

While the brief and nonpharmacological nature of this intervention makes it a promising candidate for widespread use in supporting well-being in pregnancy, a longer, more intensive intervention may be more effective in limiting excessive weight gain. When a sample has high food insecurity, as this sample did, providing healthy food directly or easier access to healthy food may improve efficacy. We are pilot testing further curriculum development to augment MMT by providing both cooking skills (hands on kitchen training) and fresh grocery bags each week, and preliminary feedback from participants has been favorable. In their evaluations, participants suggested that the MMT program could also expand mindfulness meditation and sharing time, and meet every other week until later in the pregnancy.

Recruitment and retention were difficult, requiring tremendous staff effort and devotion of study resources, which has implications for scaling to a community setting. The narrow window of eligibility for enrolling women in a group intervention during the 2nd trimester of pregnancy presents unique obstacles. Logistical issues particularly common in low-income samples, such as inflexible work schedules, lack of transportation/reliance on public transit, and need for childcare also created barriers. In order to optimize recruitment and retention efficiency for future interventions, it may be useful to more fully integrate the program with existing support programs or organizations/communities for low-income pregnant mothers.

## Conclusions

Pregnancy is a critical period for both maternal and offspring health, and there is need to reduce distress and unhealthy eating during pregnancy, particularly for women at greatest risk for high stress and excessive gestational weight gain. Through an iterative process of intervention development, we have developed a mindfulness-based program designed to reduce stress and encourage healthy weight gain that we have demonstrated is feasible to utilize with a high-stress diverse pregnant population. Partial support was found for effects of the intervention on hypothesized mechanisms of change: general mindfulness, mindful eating, acceptance, and emotion regulation. Improvements in mindfulness were correlated with decreases in stress, depression, and self-reported emotional and external eating.

By sharing our process of intervention development and initial findings regarding the mechanisms of MMT effects, we hope to make future studies in this area easier to implement. Our intervention manual is available upon request to be tailored for other populations. These findings should encourage practitioners and policymakers that even in very high risk samples, pregnancy is a window of opportunity for behavior change that can improve metabolic and mood trajectories both for women and their offspring.

## Additional files

Additional file 1: MAMAS Study Demographic Questionnaire. (PDF 568 kb)

Additional file 2: MAMAS Study Facilitator Fidelity Assessment. (PDF 292 kb)

Additional file 3: MAMAS Study Final Evaluation Questionnaire. (PDF 71 kb)

## Abbreviations

AAQ: Acceptance and action questionnaire
BMI: Body mass index
CNM: Certified Nurse Midwife
CPMC: California Pacific Medical Center
CRC: Clinical Research Center
DEBQ: Dutch eating behavior questionnaire
ERQ: Emotion Regulation Questionnaire
FFMQ: Five factor mindfulness questionnaire
FNP: Family Nurse Practitioner
IOM: Institute of Medicine
MAMAS: Maternal adiposity, metabolism, and stress
MBCT: Mindfulness-based cognitive therapy
MB-EAT: Mindfulness-Based Eating Awareness Training
MBSR: Mindfulness-based stress reduction
MEQ: Mindful eating questionnaire
MMT: Mindful moms training
ORBIT: Obesity-Related Behavioral Intervention Trials
PANAS: Positive and negative affective scale
PSS: Perceived stress scale
SHINE: Supporting Health by Integrating Nutrition and Exercise
UCSF: University of California, San Francisco
