# Supplementary material for: The mindful moms training: development of a mindfulness-based intervention to reduce stress and overeating during pregnancy
Source: BMC Pregnancy Childbirth. 2018 Jun 1;18:201. doi: 10.1186/s12884-018-1757-6 (PMC5984812; doi:10.1186/s12884-018-1757-6)
Supplement: Supplementary file 1 — MAMAS Study Demographic Questionnaire. (PDF 568 kb) [file 12884_2018_1757_MOESM1_ESM.pdf]

Screener__ __ ID: __________

☐INTERVENTION
☐ COMPARISON
☐ INELIGIBLE

MAMAS Study Screening Questionnaire

Date: _______________ Phase: ______ Wave: ______

INTRODUCTION

Phone recruitment:

Thanks for your interest in the MAMAS Study. How can I help you? Do you have any questions about the study?
[Provide brief introduction if needed:
Researchers at the University of California, San Francisco (UCSF) and California Pacific Medical Center (CPMC) are conducting a study to learn about programs to promote healthy weight gain and stress reduction during pregnancy for women who are concerned about their weight. Simple changes in diet and exercise may not be enough to help women manage their weight during pregnancy. We want to know if learning stress reduction skills in addition to receiving information about nutrition and wellness can help women achieve a healthy weight gain during pregnancy.]

In-person recruitment:

Hi, my name is ________________. I am a research assistant from the UCSF MAMAS study. I would like to tell you more about our study. Do you have a few minutes to talk?

IF YES:
Great, thank you! Researchers at the University of California, San Francisco (UCSF) and California Pacific Medical Center (CPMC) are conducting a study to learn about programs to promote healthy weight gain and stress reduction during pregnancy for women who are concerned about their weight. Simple changes in diet and exercise may not be enough to help women manage their weight during pregnancy. We want to know if learning stress reduction skills in addition to receiving information about nutrition and wellness can help women achieve a healthy weight gain during pregnancy. We are approaching pregnant women who may be eligible for the study.

IF NO:
Reason: ____
We can call to give you more information if now isn't a good time.
What's your phone number? _____________________
When is the best time to call you? DAY/TIME: ________________________

Can I ask you some questions to see if you are eligible?

IF YES:
Also, I would like to let you know that all the information you provide today will be kept confidential. You should also know that, if you are eligible and decide to participate in the study you don't have to do any part that you don't want to and you are free to drop out at any time.

IF NO:
Reason: ____
We can call to ask these questions later if now isn't a good time.
What's your phone number? _____________________
When is the best time to call you? DAY/TIME: ________________________

Screener__ __ ID: __________

CONTACT INFO

Can you please give me your contact information?

1. Name: ____
FIRST LAST

Address: ____ Email: ____________________________

____

2. Best phone number to use: ________________________ Best time to call:___________________

3. Is it OK to say we're calling from the MAMAS Study if we need to leave a message?
[1]☐ YES
[2]☐ **NO→** Specify:____

4. How did you learn about this study? ____

ELIGIBILITY QUESTIONS

Please ask all shaded questions EVEN if the individual is ineligible—unless she is not pregnant.
If eligible for and interested in COMPARISON GROUP, please complete ALL screening questions.

5. Are you currently pregnant?
[1]☐ YES
[2]☐ NO→**INELIGIBLE [STOP. "I'm sorry but the study is for women who are pregnant. Thank you so much for your time. If you know someone who is pregnant and think she may be interested in participating, please ask her to call us at 415-476-9800.''** Give the person a brochure.**]**

6. What is your date of birth?
____
MM/DD/YYYY

7. How old are you?
__________YRS **IF <18 YRS or > 45 YRS → ☐ INELIGIBLE**

8. How many weeks pregnant are you?
_________ weeks ☐ <12 weeks NOT ELIGIBLE TO ENROLL YET
☐ 20 -24 weeks → MAY BE ELIGIBLE for COMPARISON GROUP
☐ **>24 weeks → INELIGIBLE**

9. What is your estimated due date?
____
(MM/DD/YYYY)

Class start date: _________________

Eligible EDDs ________ -- _________

Screener__ __ ID: __________

10. What is your height?
____ ft. ____in

11. What was your pre-pregnancy weight (your weight <u>before</u> you became pregnant)?
________ lbs/kgs Current weight: ___________ lbs/kgs

12. BMI: _________

IF < 25 or > 41 <u>or</u> pre-pregnancy weight > 300 pounds → ☐ INELIGIBLE

13. Can you read and speak English fluently?

1☐YES
2☐ **NO → INELIGIBLE** [Unfortunately we are only able to offer our programs in English at this time.]

14. Are you currently pregnant with twins or triplets [or higher order gestation]?
1☐**YES→ INELIGIBLE**
2☐ NO

15. a. Have you had an early diabetes screening test in this pregnancy?
1☐YES
2☐ NO

b. What were the results?
1☐ Normal
2☐ **Abnormal → INELIGIBLE**
3☐ Don't know _________________________

16. Has a doctor ever told you that you have diabetes, coronary heart disease, kidney disease, cirrhosis of the liver, acute hepatitis, mental health issues, or an eating disorder?

1☐**YES→ INELIGIBLE** Please specify:____
2☐ NO

17. Have you ever been diagnosed with polycystic ovarian syndrome <u>and</u> treated with Metformin?
1☐**YES→ INELIGIBLE**
2☐ NO

18. Are you currently taking any weight loss drugs, medications for diabetes, antidepressants, antipsychotics, opiate drugs, or corticosteroids?
1☐**YES→ INELIGIBLE**
2☐ NO

19. Do you have any psychiatric, mental health, or medical diagnosis of any kind?
1☐**YES**→ Specify:____
☐ Potentially disruptive to group or difficult for participant to participate →**INELIGIBLE**
2☐ NO

20. Have you had gastric bypass surgery?
    1☐**YES→ INELIGIBLE**
    2☐ NO

21. Do you currently or have you ever had a mindfulness meditation practice with a typically sitting meditation of 20 or more minutes two or more times per week?
    1☐**YES→ INELIGIBLE**
    2☐ NO

22. What is your annual household income? In other words, how much money does everyone in your household combined make per year before taxes?

    ________ dollars

23. How many children under the age of 18 live in your home?

    _________ children

24. How many adults (age 18 or older) live in your home?

    _________ adults

25. Income to poverty ratio <500%?

    1☐YES
    2☐ **NO → INELIGIBLE**

Based on the 2012 US Poverty Guidelines
(http://aspe.hhs.gov/poverty/12poverty.shtml)
500% income to poverty ratio:
Family of 1 = $55,850
Family of 2 = $75,650
Family of 3 = $95,450
Family of 4 = $115,250
Family of 5 = $135,050
Family of 6 = $154,850
Family of 7 = $174,650
Family of 8 = $194,450
> 8, add $3,960 for each additional person

26. Are you willing to attend a 30-minute orientation session to learn more about the study?
    1☐YES
    2☐ **NO → INELIGIBLE**

We are half way through the screener. The next few questions are not eligibility questions but they give us information about the women we are reaching.

27. Are you of Hispanic or Latino origin?
    1☐YES
    2☐ NO

28. Which race best describes you? (Check all that apply)

    1☐ African American/Black
    2☐American Indian/Native American
    3☐ Alaskan Native
    4☐ Asian
    5☐ Caucasian/White
    6☐ Pacific Islander
    7☐ Other: __________________

29. What is your current relationship status? (Mark one)

[1]☐ Single or casually dating
[2]☐ In a committed relationship or engaged
[3]☐ Living in a marriage-like relationship
[4]☐ Presently married
[5]☐ Separated
[6]☐ Divorced
[7]☐ Widowed

30. What is the highest grade in school you finished? (Mark one)

[1]☐ Did not finish elementary school
[2]☐ Finished middle school (8th grade)
[3]☐ Finished some high school
[4]☐ High school graduate or G.E.D.
[5]☐ Vocational or training school after high school
[6]☐ Some College or Associate Degree
[7]☐ College graduate or Baccalaureate Degree
[8]☐ Masters (MS) or Doctoral Degree (PhD, MD, JD, etc)

♦ If the person is INELIGIBLE for intervention and comparison groups, STOP here. ♦

Do not ask the remaining questions.

31. Which of these categories best describes your personal income (not the income of your household, but your own income) for the past 12 months? This should include (before taxes) from all sources, wages, veteran's benefits, help from relatives, rent from properties and so on. (Adapted from CARDIA)

[1]☐Less than $5,000
[2]☐$5,000 through $11,999
[3]☐$12,000 through $15,999
[4]☐$16,000 through $24,999
[5]☐$25,000 through $34,999
[6]☐$35,000 through $49,999
[7]☐$50,000 through $74,999
[8]☐$75,000 through $99,999
[9]☐$100,000 through $149,999
[10]☐$150,000 through $199,999
[11]☐$200,000 or more

32. Have you ever had a miscarriage after 12 weeks of pregnancy?
 1☐YES
 2☐ NO

33. Have you had a previous spontaneous preterm birth (before 37 weeks of pregnancy)?
 1☐YES
 2☐ NO

34. Is this your first child?
 1☐ YES [SKIP TO Q38]
 2☐ NO

35. How many children have you given birth to?
 _________ children

36. How much weight did you gain with your other pregnancies? [Ask for total weight gain in each pregnancy.]

 a. 1st pregnancy __________ lbs/kgs
 b. 2nd pregnancy __________ lbs/kgs
 c. 3rd pregnancy __________ lbs/kgs
 d. 4th pregnancy __________ lbs/kgs
 e. 5th pregnancy __________ lbs/kgs
 f. 6th pregnancy __________ lbs/kgs

37. Have you had a previous low birth weight infant (less than 5 ½ pounds or 2500 grams)?
 1☐YES
 2☐ NO

38. a. Where are you going for prenatal care?
 ☐ __________________________________ (CODE: _______)
 2☐ No prenatal care provider → **Give list of providers and other services**

 b. Do you plan to have your baby there?
 1☐YES
 2☐ NO → Where do you plan to deliver your baby? ________________________ CODE :(_______)

39. How was your due date determined?
 ☐ LMP
 ☐ ULTRASOUND Date of u/s: ___________

40. What was the FIRST day of your last menstrual period?
 ____
 (MM/DD/YYYY)

41. Are you participating in Centering, Black Infant Health, or any other [intervention] program?
   - ☐ Yes, Centering (Location: _________________________________)
   - ☐ Yes, Black Infant Health
   - ☐ Yes, Other: ____
   - ☐ NO

********* **Please complete the following items. Do NOT ask the potential participant.** **********

In your contact with the potential participant, did she demonstrate any of the following items that would lead her to be inappropriate for this study or for the group setting?

☐ Distractibility to the point that the interviewer is unable to keep the individual on task even when redirected.

☐ Lack of comprehension to the point that the individual does not understand the questions asked.

☐ Dominating to the point that the interviewer is unable to ask questions, indicating that the individual would be hard to manage in a group setting.

☐ Odd thinking and speech (i.e. such that the interviewer can't understand the words the individual uses, words are made up).

☐ Other difficult personality or cognitive functioning concerns. Please describe_______________________
____

If any of the above items are checked, please tell the potential participant:
"Thank you for your time. I will review this with the study team and if you are potentially eligible, I will call you back to let you know."

A study investigator must be consulted to determine eligibility.

Name of study clinician contacted: _____________________________________ Date contacted: _________

☐ ELIGIBLE FOR INTERVENTION GROUP
☐ ELIGIBLE FOR COMPARISON GROUP ONLY } ASK ALL SCREENING QUESTIONS

☐ INELIGIBLE: ____

Screener__ __ ID: __________

IF ELIGIBLE FOR COMPARISON GROUP:

"I'm sorry, but you are too far along in your pregnancy to participate in the study classes but you appear to be eligible to participate in another part of the study. We are inviting women who are between 20 and 28 weeks pregnant to enroll in a comparison group. If you decide to participate, we will ask you to complete study questionnaires when you first enroll and again 8-10 weeks later. Finally we will ask you to give us permission to review your prenatal and birth hospitalization records after you have your baby. The information from women in the comparison group will help us to better understand if and how the classes might benefit pregnant women. You would be paid up to $50 for participating in the comparison group. Do you have any questions? Would you like to participate in the comparison group?

☐ YES
☐ DECLINES TO PARTICIPATE (Specify reason: ____
____)

IF ELIGIBLE FOR THE INTERVENTION GROUP:

"You appear to be eligible. As I mentioned earlier, the purpose of this study is to help us learn more about programs to promote healthy weight gain and stress reduction during pregnancy for women who are concerned about their weight. If you decide to participate in the study, you will attend Mindful Moms classes that meet for 2 hours once a week, for 8 weeks at St. Luke's Hospital [at 3555 Cesar Chavez in San Francisco]. The classes will teach women ways to decrease stress and have a healthy relationship with food through increased awareness of your real level of hunger and fullness. The information you receive can help you have a healthier pregnancy.

You will also be asked to complete questionnaires and have a physical assessment before and after the 8 week program, at 32-34 weeks of pregnancy, and at 3 and 9 months after your baby is born. We want to understand if and how learning mindfulness-based stress reduction skills in the Mindful Moms classes might have additional benefits for pregnant women.

Finally, we realize that your time is valuable so you will be paid up to $760 for participating in the MAMAS Study.

I know I've just given you a lot of information. Do you have any questions?

Please note questions/concerns:
____
____
____

☐ DECLINES TO PARTICIPATE (Specify reason: ____
____)

Screener__ __ ID: __________

IF STILL INTERESTED:
If you think you would like to participate, the first step is to attend an orientation session to learn more about the study, get answers to any questions you have, and have a chance to discuss the pros and cons of participating.
The next orientation session will be held on :__________________(date/time/location). Can you make this date?

☐YES	☐ NO [Please note availability and offer individual orientation session if needed] ____

After attending the orientation session, if you decide to enroll in the study, we will schedule an appointment for you at the UCSF Clinical Research Center at Parnassus for an initial assessment. The assessment takes 2-3 hours and includes body measurements, a blood draw, an ultrasound exam, and questionnaires.

I'm so glad that you're interested in the study. Here's some additional information and my card in case you think of any more questions.
