# Supplementary material for: The mindful moms training: development of a mindfulness-based intervention to reduce stress and overeating during pregnancy
Source: BMC Pregnancy Childbirth. 2018 Jun 1;18:201. doi: 10.1186/s12884-018-1757-6 (PMC5984812; doi:10.1186/s12884-018-1757-6)
Supplement: Supplementary file 2 — MAMAS Study Facilitator Fidelity Assessment. (PDF 292 kb) [file 12884_2018_1757_MOESM2_ESM.pdf]

1. Which audio recording are you coding? (please use the exact title from the Dropbox audio file, e.g. May 8 2012 Session 1)

Session 1 - Introductions (30 Minutes)

2. Did facilitators provide women with logistical information, such as restroom locations?

- Not at all
- Yes, but not complete
- Yes, they provided complete information

Other (please specify)

3. Did facilitators introduce themselves briefly, briefly describing their training, personal experience with pregnancy/children, and personal experience with stress/weight/exercise issues?

- Not at all
- Yes, but not complete
- Yes, they provided complete information

Other (please specify)

4. Did facilitators allow women to introduce themselves very briefly, limiting each to two minutes and putting questions on the board?

- No, not at all
- Yes, but not completely
- Yes, complete information was provided

Other (please specify)

Session 1 - Welcome and Overview (10 Minutes)

5. Did instructors give a brief summary of Mindful Moms Training (explain that they will gain practices and skills for dealing with stress, and for healthy eating and staying physically active during pregnancy to provide a healthy environment for the baby to grow in)?

- ◯ No, not at all
- ◯ Yes, but not completely
- ◯ Yes, complete information was provided

Other (please specify)

6. Did instructors explain why this course is so important? (E.g. gaining too much weight during pregnancy, and being too stressed out, can increase the risk of the baby growing up overweight, it can increase risks for difficult childbirth, more pain and discomfort during pregnancy, and a hard time returning to your prepregnancy weight)

- ◯ No, not at all
- ◯ Yes, but not completely
- ◯ Yes, complete information was provided

Other (please specify)

7. Did instructors explain why this was not a diet and exercise program (it doesn't focus only on eating and exercise but also on stress reduction techniques, and the training will increase the pleasant and joyful parts of being pregnant and becoming a mom and will connect participants more with their new baby)?

- ◯ No, not at all
- ◯ Yes, but not completely
- ◯ Yes, complete information was provided

Other (please specify)

8. Did facilitators ask women to pull out their binders and note on the calendar any weeks that will be missed due to holidays and point out instructor/assistant contact information, and ask women to please contact the instructor/assistant if they cannot attend classes?

- No, not at all
- Yes, but not completely
- Yes, complete information was provided

Other (please specify)

9. Did instructors go over the general format of the sessions (that they will meet for eight weeks, two hours per week,and instruct wormen to make every effort to be on time)?

- No, not at all
- Yes, but not completely
- Yes, complete information was provided

Other (please specify)

10. Did they review the ground rules one by one and ask each participant to sign one copy to turn in, and keep one copy?

- No, not at all
- Yes, but not completely
- Yes, complete information was provided

Other (please specify)

11. Were the topics of confidentiality and limits of confidentiality discussed?

- ◯ No, not at all
- ◯ Yes, but not completely
- ◯ Yes, complete information was provided

Other (please specify)

What are we asking you to do (15 min)

12. Did facilitators go over the three commitments handout in detail. The three commitments are:
1)Eating mindfully (mindful awareness of hunger and fullness, of how the food tastes, and of food choices)
2)Move Your Body actively and with awareness
3)Breathing With Awareness

- ◯ No, not at all
- ◯ Yes, but not completely
- ◯ Yes, complete information was provided

Other (please specify)

13. Did the instructor discuss “inner” wisdom versus “outer” knowledge in regard to mindful eating?

- ◯ No, not at all
- ◯ Yes, but not completely
- ◯ Yes, complete information was provided

Other (please specify)

14. Did facilitators ask woman to commit to at least one mindful awareness practice each day during the week (e.g. using the recorded guidance, taking a mindful shower, sitting quietly)?
And share that the homework in between sessions is to

1) Practice Mindful Healthy Eating
2) Actively move your body for 20 minutes each day.
3) Mindful Awareness/Stress Reduction Practices
4) Keep daily records
5) Read Assigned Readings for each week

- ◯ No, not at all
- ◯ Yes, but not completely
- ◯ Yes, complete information was provided

Other (please specify)

[ ]

Mindful Exercises/Plan (45 min)

15. Did facilitator do a mindful eating exercise with a tangerine or a raison, with a guided meditation and discussion (20 min)?

- No, not at all
- Yes, but not completely
- Yes, complete information was provided

Other (please specify)

16. Did facilitators go over the EAT MORE/eat less plan in detail? (10 min)

- No, not at all
- Yes, but not completely
- Yes, complete information was provided

Other (please specify)

17. Did the facilitators inform participants that the Mindful Movement will begin next week and did they do a 10 Minute mindful awareness of breathing exercise?

- No, not at all
- Yes, but not completely
- Yes, complete information was provided

Other (please specify)

18. Do the facilitators mention the baby at least a few times in each guided period of practice whether in movement, breathing, body sensations, or open awareness?

- ○ No, not at all
- ○ Yes, but not completely
- ○ Yes, complete information was provided

Other (please specify)

Reminder and Closing (15 Min)

19. Did facilitators review the three commitments? Asking the participants if they remember what they were and call on a few people to state them out loud?

- ○ No, not at all
- ○ Yes, but not completely
- ○ Yes, complete information was provided

Other (please specify)

20. Was there a closing silent sitting practice that ended with a loving-kindness meditation where the following phrases are repeated internally:
• May I be safe.
• May I be happy.
• May I be healthy and strong.
• May I live with ease.

(Then repeating the intention replacing "I" with “we”)?

- ○ No, not at all
- ○ Yes, but not completely
- ○ Yes, complete information was provided

Other (please specify)

1. Which audio recording are you coding? (please use the exact title from the Dropbox audio file, e.g. May 8 2012 Session 1)

Session 2- Mindful Movement and Check-in (35-40 Min)

2. Did instructors ask the participants to turn in their Daily Record? And if they did not complete or bring their Daily Record, ask them to sit down for a few moments and complete it from memory?

- ◯ No, not at all
- ◯ Yes, but not completely
- ◯ Yes, complete information was provided

Other (please specify)

3. Did facilitators remind women not stretch beyond their comfort level, and to check with a physician if they have any questions about their ability to participate in any of the exercises?

- ◯ No, not at all
- ◯ Yes, but not completely
- ◯ Yes, complete information was provided

Other (please specify)

4. Did facilitators do a mindful movement session starting with a body scan and focusing on noticing and accepting whatever they experience?

- ◯ No, not at all
- ◯ Yes, but not completely
- ◯ Yes, complete information was provided

Other (please specify)

5. Did facilitators check in with women if they moved their bodies this week?

- No, not at all
- Yes, but not completely
- Yes, complete information was provided

Other (please specify)

6. Did participants briefly check-in (about one minute each) about how the eat more/eat less plan went this last week?

- No, not at all
- Yes, but not completely
- Yes, complete information was provided

Other (please specify)

7. Did facilitators review the EAT MORE/eat less Handout in detail and go over each recommendation and ask the class to give a couple of examples of each one?

- No, not at all
- Yes, but not completely
- Yes, complete information was provided

Other (please specify)

8. Did facilitators brainstorm with women using questions such as:
What of this are you already doing? What might be easy? What might be hard? What could be a way to do this that is in your budget?

- ◯ No, not at all
- ◯ Yes, but not completely
- ◯ Yes, complete information was provided

Other (please specify)

Session 2- Control vs. Mindfulness (15 minutes)

9. Did facilitator check in with women about if there were able to practice mindful awareness this week (with or without the CD)?

- No, not at all
- Yes, but not completely
- Yes, complete information was provided

Other (please specify)

10. Did facilitators ask participants what the three commitments were and write them on the board?

- No, not at all
- Yes, but not completely
- Yes, complete information was provided

Other (please specify)

11. Did facilitators ask participants if they have ever tried to change their eating or weight in the past? And did they explain the difference between controlling and being mindful?

- No, not at all
- Yes, but not completely
- Yes, complete information was provided

Other (please specify)

12. Did instructors use the Finger Cuffs exercise to demonstrate how resisting experiences and fighting against them is futile? Did they have a discussion about the Finger Trap exercise?

- ◯ No, not at all
- ◯ Yes, but not completely
- ◯ Yes, complete information was provided

Other (please specify)

13. Did facilitators lead the chocolate cake/favorite food thought exercise with participants to illustrate the concept of what you resist persists?

- ◯ No, not at all
- ◯ Yes, but not completely
- ◯ Yes, complete information was provided

Other (please specify)

14. Did facilitators explain what mindfulness is (moment-to-moment, nonjudgmental awareness of our experience)?

- ◯ No, not at all
- ◯ Yes, but not completely
- ◯ Yes, complete information was provided

Other (please specify)

15. Did facilitators do the acceptance exercise on the board, asking women for words to describe acceptance? Did they explain that acceptance is not passive but a willingness to meet things as they are?

- No, not at all
- Yes, but not completely
- Yes, complete information was provided

Other (please specify)

16. Did facilitators explain that acceptance is facing situations exactly as they are, being willing to see them clearly, and making decisions about how to respond to them as they are? Did they also give an example of a acceptance in everyday life such as a car towed, a women being in an abusive relationship, or an alcoholic?

- No, not at all
- Yes, but not completely
- Yes, complete information was provided

Other (please specify)

Mindful Awareness of Breathing and Mindful Eating (40 min)

17. Did instructors lead a 10 minute mindful awareness breathing exercise with a discussion after?

- ◯ No, not at all
- ◯ Yes, but not completely
- ◯ Yes, complete information was provided

Other (please specify)

18. Did instructors ask the women how practicing with the meditation CD was this week? Did they remind the women to practice with the CD in the following week?

- ◯ No, not at all
- ◯ Yes, but not completely
- ◯ Yes, complete information was provided

Other (please specify)

19. Did instructors lead the mindful eating exercise with crackers and cheese with a discussion at the end?

- ◯ No, not at all
- ◯ Yes, but not completely
- ◯ Yes, complete information was provided

Other (please specify)

20. Did instructors review the three commitments (Mindful eating, move my body, breathe) and review the homework for the week?

- No, not at all
- Yes, but not completely
- Yes, complete information was provided

Other (please specify)

21. Did facilitators lead a silent closing practice, ending with loving-kindness meditation where the following phrases are repeated internally:

• May I be safe.
• May I be happy.
• May I be healthy and strong.
• May I live with ease.

(Then repeating the intention replacing "I" with “we”)?

- No, not at all
- Yes, but not completely
- Yes, complete information was provided

Other (please specify)

1. Which audio recording are you coding? (please use the exact title from the Dropbox audio file, e.g. May 8 2012 Session 1)

Session 3- Movement and Check-In (30 Minutes)

2. Did facilitators ask participants to turn in their Daily Record and if they did not complete or bring their Daily Record, did they give them a few moments to complete it from memory?

- ◯ No, not at all
- ◯ Yes, but not completely
- ◯ Yes, complete information was provided

Other (please specify)

3. Before the mindful movement exercise, did facilitators remind women to not stretch beyond their comfort level, and to check with a physician if they have any questions about their ability to participate in any of the exercises?

- ◯ No, not at all
- ◯ Yes, but not completely
- ◯ Yes, complete information was provided

Other (please specify)

4. In the mindful movement series, did facilitators instruct women to observe as nonjudgmentally as possible by noting, naming, and witnessing tension or other physical sensations. (e.g. "Just notice the sensations in your body. Try simply noting, or naming sensations that arise – without calling them 'good' or 'bad,' or trying to change them. Let them be as they are.")

- ◯ No, not at all
- ◯ Yes, but not completely
- ◯ Yes, complete information was provided

Other (please specify)

5. Did facilitators write down check-in questions on the board and instruct participants to find a partner and discuss the questions for five minutes each (ringing a bell to let them know when it is time switch partners)?

- ◯ No, not at all
- ◯ Yes, but not completely
- ◯ Yes, complete information was provided

Other (please specify)

6. Did facilitators lead participants to come back into the large group and share what some of the common themes in terms of challenges and new learnings or successes? And did they engage in problem-solving and brainstorming about ways to integrate homework into life? (Using inquiry rather than suggestions or advice, e.g. What is the primary barrier to practice?)

- ◯ No, not at all
- ◯ Yes, but not completely
- ◯ Yes, complete information was provided

Other (please specify)

7. Did facilitators lead members through a hunger awareness meditation?

- ◯ No, not at all
- ◯ Yes, but not completely
- ◯ Yes, complete information was provided

Other (please specify)

8. Did facilitators lead members through a hunger awareness meditation?

- ◯ No, not at all
- ◯ Yes, but not completely
- ◯ Yes, complete information was provided

Other (please specify)

Session 3- Outer Knowledge: Introduction to Proportions and Portions (20 Minutes)

9. Did instructors go over the Plate Method in detail? (½ veggies, ¼ starches, ¼ protein, and just a little bit of fats, sweets)

- ◯ No, not at all
- ◯ Yes, but not completely
- ◯ Yes, complete information was provided

Other (please specify)

10. Did instructors give pointers on things such as how to estimate portion sizes (by using the palm of your hand) and reminding women that while pregnant, eating small meals throughout the day is ideal, and give helpful suggestions such as using smaller plates?

- ◯ No, not at all
- ◯ Yes, but not completely
- ◯ Yes, complete information was provided

Other (please specify)

Session 3- Mindfulness/Acceptance Concept: The Observing Self (15 minutes)

11. Did facilitators talk about awareness as a component of everything we experience and share the metaphor of the balloon in the breadbox?

- ○ No, not at all
- ○ Yes, but not completely
- ○ Yes, complete information was provided

Other (please specify)

12. Did instructors explain the sky mind? (Our awareness is like a sky, where thoughts, feelings, and sensations are all like clouds, that arise and then pass away.)

- ○ No, not at all
- ○ Yes, but not completely
- ○ Yes, complete information was provided

Other (please specify)

13. Did facilitator explain the Chessboard Metaphor? (Every thought or feeling and experience you've had is like a piece on your chessboard, and your awareness is like the chessboard itself that has no boundaries)

- ○ No, not at all
- ○ Yes, but not completely
- ○ Yes, complete information was provided

Other (please specify)

Mindful Awareness Practice (10 minutes)

14. Did facilitators guide a mindful awareness of breathing, focusing specifically on the observing self, the internal witness who views experiences with curiosity, interest, and compassion?

- ◯ No, not at all
- ◯ Yes, but not completely
- ◯ Yes, complete information was provided

Other (please specify)

15. Did facilitators mention the baby at least a few times within this guided practice?

- ◯ No, not at all
- ◯ Yes, but not completely
- ◯ Yes, complete information was provided

Other (please specify)

16. Did facilitators lead a discussion of the mindfulness exercise giving them a list of emotions and sensations from the handouts to help them to better describe their experiences? Did facilitators ask how this experience of observing can apply to healthy eating?

- ◯ No, not at all
- ◯ Yes, but not completely
- ◯ Yes, complete information was provided

Other (please specify)

Hunger Awareness (20 minutes)

17. Did facilitators lead a discussion on what hunger is and talk about physical hunger vs. psychological hunger and when to eat?

- ◯ No, not at all
- ◯ Yes, but not completely
- ◯ Yes, complete information was provided

Other (please specify)

18. Did facilitators lead the hunger awareness meditation asking women to mindfully rate their hunger on a scale from 1 to 10 and exploring the feelings and sensations that lead them to picking their number?

- ◯ No, not at all
- ◯ Yes, but not completely
- ◯ Yes, complete information was provided

Other (please specify)

19. Did instructors draw the 10 point Hunger Scale on the board and have participants report their numbers and best describe the physical sensations that led them to assign the given number? And did they make a distinction between physical sensations and emotional feelings about hunger?

- ◯ No, not at all
- ◯ Yes, but not completely
- ◯ Yes, complete information was provided

Other (please specify)

20. Did facilitators lead discussion on how physical hunger is different from psychological hunger? Did they ask what emotions lead participants to overeat?

- ◯ No, not at all
- ◯ Yes, but not completely
- ◯ Yes, complete information was provided

Other (please specify)

Session 3- Review Commitments and Closing (20 Min)

21. Did facilitators review the three commitments having everyone say the commitments aloud (mindful eating, move my body, and breath) and remind them of their homework for the week?

- ○ No, not at all
- ○ Yes, but not completely
- ○ Yes, complete information was provided

Other (please specify)

22. Was there a closing silent sitting practice that ended with a loving-kindness meditation where the following phrases are repeated internally:

• May I be safe.
• May I be happy.
• May I be healthy and strong.
• May I live with ease.

(Then repeating intention replacing "we” for "I")?

- ○ No, not at all
- ○ Yes, but not completely
- ○ Yes, complete information was provided

Other (please specify)

1. Which audio recording are you coding? (please use the exact title from the Dropbox audio file, e.g. May 8 2012 Session 1)

Movement and Check-In (30 Minutes)

2. Did facilitators ask participants to turn in their Daily Record and if they did not complete or bring their Daily Record, did they give them a few moments to complete it from memory?

- ○ No, not at all
- ○ Yes, but not completely
- ○ Yes, complete information was provided

Other (please specify)

3. Did facilitators lead a Mindful Walking exercise instructing women to keep their awareness on the sensations in their bodies, gently bringing it back when their mind has wandered?

- ○ No, not at all
- ○ Yes, but not completely
- ○ Yes, complete information was provided

Other (please specify)

4. Did participants check-in with each other in dyads about how using the Proportions and Portions went and what they noticed about how much protein vs. veggies vs. carbs/breads they typically eat? (Allowing each women to talk for 5 minutes and ringing a bell to notify when to switch)

- ○ No, not at all
- ○ Yes, but not completely
- ○ Yes, complete information was provided

Other (please specify)

5. In the large group did women check in about the Three Commitments: Mindful Eating, Move My Body, Breathe?

- ◯ No, not at all
- ◯ Yes, but not completely
- ◯ Yes, complete information was provided

Other (please specify)

Introduction to Stomach Fullness (20 minutes)

6. Did instructors talk about “satiety” signals and ask the group what these are and ask what some other reasons are that they continue to eat when they have had enough?

- No, not at all
- Yes, but not completely
- Yes, complete information was provided

Other (please specify)

7. Did facilitators put the fullness scale on the board (with 1 as not full at all, and 10 as completely full, asking participants where moderately full would be and what physical signals represent it)? Did they also give good ways of conceptualizing when they are full enough (e.g. that they could still exercise moderately after eating and feel comfortable)?

- No, not at all
- Yes, but not completely
- Yes, complete information was provided

Other (please specify)

8. Did facilitator lead the Stomach Fullness Meditation twice, (one time before drinking a couple of glasses of water and once after drinking the water)?

- No, not at all
- Yes, but not completely
- Yes, complete information was provided

Other (please specify)

9. Did instructors lead a discussion after Fullness Meditation and emphasize that everyone will have different feelings, and that there is no right or wrong answers? And did they ask what types of situations, feelings or thoughts might get in the way of paying attention to fullness and encourage everyone to try to notice these experiences next week?

- No, not at all
- Yes, but not completely
- Yes, complete information was provided

Other (please specify)

10. Did instructors lead a discussion addressing the relationship between experiences of hunger decreasing as you eat, and experiences of fullness increasing and draw an example of how to use the scales on the board? Did they encourage individuals to observe their experiences during the week in regard to feeling physically hungry and physically full, and the extent to which they overlap or not?

- No, not at all
- Yes, but not completely
- Yes, complete information was provided

Other (please specify)

Mindfulness/Acceptance Concept: Train of Thought (15 minutes)

11. Did facilitators include these main points into the Train of Thought portion of the class:

• Thoughts are not Facts: Cognitive distortions – the mind creates stories about the meaning of every situation. We mistake these stories as truth.
• The mind simply creates stories about what it is perceiving through filters of expectations, memories and feelings.
• Evaluating experience – By habitually evaluating all our experiences and getting invested in those evaluations, we produce struggle to make what we don't like go away and to hold onto what we want more of.
• The mind looks for what's not working, and is in constant comparison with others (or who we think we should be).
• Don't believe everything you think – use the mind as one source of information.
• Thank the mind for sharing, and then rest in your larger awareness.

◯ No, not at all

◯ Yes, but not completely

◯ Yes, complete information was provided

Other (please specify)

12. Did the facilitator give an example of how our minds believe previous assumptions and think that they are truth when they are not (e.g what color is a Yield sign exercise)?

◯ No, not at all

◯ Yes, but not completely

◯ Yes, complete information was provided

Other (please specify)

13. Did facilitators give the Two Computer Metaphor?

- ○ No, not at all
- ○ Yes, but not completely
- ○ Yes, complete information was provided

Other (please specify)

Mindful Awareness of Thoughts (10 Minutes)

14. Did facilitators start the mindful awareness period with attention to breathing, and make sure that participants assumed a wakeful, alert, rested posture?

- No, not at all
- Yes, but not completely
- Yes, complete information was provided

Other (please specify)

15. Did facilitators guide the mindful awareness of thoughts, having participants bring their attention to their thoughts and use the following metaphors to help explain; (train metaphor, CNN announcer metaphor, and the metaphor of the bullhorn vs. the radio playing)?

- No, not at all
- Yes, but not completely
- Yes, complete information was provided

Other (please specify)

16. Did facilitators mention the baby at least a few times within this guided practice?

- No, not at all
- Yes, but not completely
- Yes, complete information was provided

Other (please specify)

17. Did facilitators lead a discussion of the mindfulness exercise?

- No, not at all
- Yes, but not completely
- Yes, complete information was provided

Other (please specify)

Behavioral Commitments, Homework & Closing (15 minutes)

18. Did facilitators review the three commitments and ask people what gets in the way of being able to do any of them and help them troubleshoot (letting them come up with their own solutions rather than the facilitator giving advice)?

- No, not at all
- Yes, but not completely
- Yes, complete information was provided

Other (please specify)

19. Did facilitators review the homework assignments for the upcoming week?

- No, not at all
- Yes, but not completely
- Yes, complete information was provided

Other (please specify)

20. Was there a closing silent sitting practice that ended with a loving-kindness meditation where the following phrases are repeated internally:
• May I be safe.
• May I be happy.
• May I be healthy and strong.
• May I live with ease.

(Repeating the intention again inserting "we" instead of "I")

- No, not at all
- Yes, but not completely
- Yes, complete information was provided

Other (please specify)

1. Which audio recording are you coding? (please use the exact title from the Dropbox audio file, e.g. May 8 2012 Session 1)

Check-In (15 Minutes)

2. Did facilitators ask participants to turn in their Daily Record and if they did not complete or bring their Daily Record, did they give them a few moments to complete it from memory?

- No, not at all
- Yes, but not completely
- Yes, complete information was provided

Other (please specify)

3. Did participants check in as a large group by answering the questions: What are you thinking? What are you feeling? What is at least one body sensation you are experiencing right now? Are you aware of the part of yourself that is aware of these thoughts, feelings and sensations?

- No, not at all
- Yes, but not completely
- Yes, complete information was provided

Other (please specify)

Qualities of Mindful Awareness (20 minutes)

4. Did instructors review what they have covered so far in previous classes about mindfulness (just allowing things to be as they are, acceptance etc.)?

- ○ No, not at all
- ○ Yes, but not completely
- ○ Yes, complete information was provided

Other (please specify)

5. Did instructors go over the qualities of mindful awareness? (Non-striving, Beginner’s Mind, Present-Moment Focus)

- ○ No, not at all
- ○ Yes, but not completely
- ○ Yes, complete information was provided

Other (please specify)

Mindful Movement (20 minutes)

6. Did instructors facilitate a mindful walk outside and remind women to be aware of their body sensations, stay open-minded and accepting of whatever happens, be curious, practice non-striving, and keep awareness and attention on the present moment?

- ○ No, not at all
- ○ Yes, but not completely
- ○ Yes, complete information was provided

Other (please specify)

7. Did the facilitator lead a discussion about the Mindful Walk?

- ○ No, not at all
- ○ Yes, but not completely
- ○ Yes, complete information was provided

Other (please specify)

How Eating and Foods Relate to Stress (20 minutes)

8. Did facilitators begin with a discussion about what are the types of foods participants typically eat when under stress and write the examples of foods up on the board?

- No, not at all
- Yes, but not completely
- Yes, complete information was provided

Other (please specify)

9. Did facilitators describe the impact of sugar, fat, and alcohol on the physical stress response?

- No, not at all
- Yes, but not completely
- Yes, complete information was provided

Other (please specify)

Emotional Eating and Other Triggers in Dyads (15 Minutes)

10. Did women get into dyads to complete the Emotional Eating and Other Triggers Worksheet with one another, noticing what triggers can make them want to eat and what they might be able to do instead and then return to the group to share some themes?

- No, not at all
- Yes, but not completely
- Yes, complete information was provided

Other (please specify)

11. Did facilitators guide a Mindful Eating Exercise with a possible "problem" food such as Chocolate?

- No, not at all
- Yes, but not completely
- Yes, complete information was provided

Other (please specify)

12. Did instructors lead a brief discussion of the Mindful Eating Experience and encourage everyone to try it at home and experiment with different foods, with different amounts, and with different levels of hunger? Did they also ask what types of situations, feelings or thoughts might get in the way of paying attention to taste satisfaction – or to continuing to eat when something no longer tastes as good?

- No, not at all
- Yes, but not completely
- Yes, complete information was provided

Other (please specify)

Mindful Awareness of Body Emotions, Moods, Stress Level (10-15 Minutes)

13. Did instructors facilitate a mindful awareness of body sensations, thoughts, and feelings, focusing primarily on awareness of emotions, feelings, moods (beginning with mindful awareness of breathing and including the baby in each mindful awareness practice)?

- No, not at all
- Yes, but not completely
- Yes, complete information was provided

Other (please specify)

14. Did participants discuss their experience during mindful awareness practices?

- No, not at all
- Yes, but not completely
- Yes, complete information was provided

Other (please specify)

Pot Luck, Behavioral Commitments, & Closing (20 minutes)

15. Did facilitators inform women about the upcoming pot luck and about what to bring to the pot luck?

- No, not at all
- Yes, but not completely
- Yes, complete information was provided

Other (please specify)

16. Did facilitators review the three commitments?

- No, not at all
- Yes, but not completely
- Yes, complete information was provided

Other (please specify)

17. Did facilitators review the homework assignments for the upcoming week?

- No, not at all
- Yes, but not completely
- Yes, complete information was provided

Other (please specify)

18. Was there a closing silent sitting practice that ended with a loving-kindness meditation where the following phrases are repeated internally:
• May I be safe.
• May I be happy.
• May I be healthy and strong.
• May I live with ease.

(Repeating the intention again inserting "we" instead of "I")?

- ◯ No, not at all
- ◯ Yes, but not completely
- ◯ Yes, complete information was provided

Other (please specify)

1. Which audio recording are you coding? (please use the exact title from the Dropbox audio file, e.g. May 8 2012 Session 1)

Movement and Check-In (40 Minutes)

2. Did facilitators ask participants to turn in their Daily Record and if they did not complete or bring their Daily Record, did they give them a few moments to complete it from memory?

- No, not at all
- Yes, but not completely
- Yes, complete information was provided

Other (please specify)

3. Did instructors remind women to not stretch beyond their comfort level, and to check with a physician if they have any questions about their ability to participate in any of the exercises?

- No, not at all
- Yes, but not completely
- Yes, complete information was provided

Other (please specify)

4. During this mindful movement series did the instructors focus on bringing women into the present moment with the practice, gently helping women to rest their attention in each moment?

- No, not at all
- Yes, but not completely
- Yes, complete information was provided

Other (please specify)

5. Did each women answer one of the following questions during check in:
- Did you practice any of the qualities we used in our mindful walk last week?
- How about taste? Did you notice anything this week about paying attention to how things taste, and when the flavor disappears?
- You’ve now learned the three main aspects of our Outer Wisdom for this program: What to Eat, How Much to Eat, and How and When to Eat. How’s it going??

- ○ No, not at all
- ○ Yes, but not completely
- ○ Yes, complete information was provided

Other (please specify)

Present Moment Focus/Awareness of present moment (20 minutes)

6. Did facilitators talk about present moment focus and encourage a shift that balances from being eighty percent of attention being to the future and the past to eighty percent of that attention being here in this present moment where the action really is?

- ○ No, not at all
- ○ Yes, but not completely
- ○ Yes, complete information was provided

Other (please specify)

7. Did instructors facilitate a mindful awareness of the present moment including mentioning the baby in the mindful awareness practice?

- ○ No, not at all
- ○ Yes, but not completely
- ○ Yes, complete information was provided

Other (please specify)

Mindful Eating: Cookies and Chips (15 minutes)

8. Did facilitators discuss the Mindful Moms Check In cue cards and remind the participants that they recommend they do this before every snack and meal?

- ○ No, not at all
- ○ Yes, but not completely
- ○ Yes, complete information was provided

Other (please specify)

9. Did facilitators lead an awareness exercise of hunger and satiety with chips and cookies?

- ○ No, not at all
- ○ Yes, but not completely
- ○ Yes, complete information was provided

Other (please specify)

10. Did instructors lead a discussion of the Mindful Eating Experience and encourage everyone to try at home and experiment with different foods and different levels of hunger? Did they also talk about the “outer wisdom” of how many calories/ingredients are in different foods?

- ○ No, not at all
- ○ Yes, but not completely
- ○ Yes, complete information was provided

Other (please specify)

Behavioral Commitments, Pot Luck Planning, & Closing (30 minutes)

11. Did facilitators point to the handouts on exercise for this week and remind women to try to get at least 20 minutes of good vigorous movement each day?

- No, not at all
- Yes, but not completely
- Yes, complete information was provided

Other (please specify)

12. Did facilitators review the three commitments?

- No, not at all
- Yes, but not completely
- Yes, complete information was provided

Other (please specify)

13. Did facilitators remind mothers that the following week there will be a pot luck and have them discuss in pairs what they have in mind to bring?

- No, not at all
- Yes, but not completely
- Yes, complete information was provided

Other (please specify)

14. Did facilitators encourage participants to bring foods that will not need much preparation at the time of the potluck and inform the group about the approximate time of the meal during the session, so those with blood sugar problems, etc., can plan accordingly?

- No, not at all
- Yes, but not completely
- Yes, complete information was provided

Other (please specify)

15. Did facilitators review the homework assignments for the upcoming week?

- No, not at all
- Yes, but not completely
- Yes, complete information was provided

Other (please specify)

16. Was there a closing silent sitting practice that ended with a loving-kindness meditation where the following phrases are repeated internally:
• May I be safe.
• May I be happy.
• May I be healthy and strong.
• May I live with ease.

(Repeating again inserting the word "we" in place of "I")?

- No, not at all
- Yes, but not completely
- Yes, complete information was provided

Other (please specify)

1. Which audio recording are you coding? (please use the exact title from the Dropbox audio file, e.g. May 8 2012 Session 1)

Movement and Check-In (30 Minutes)

2. Did facilitators ask participants to turn in their Daily Record and if they did not complete or bring their Daily Record, did they give them a few moments to complete it from memory?

- No, not at all
- Yes, but not completely
- Yes, complete information was provided

Other (please specify)

3. During the Mindful Movement series did instructors use less facilitation this week and see if participants can do the series from memory, providing guidance for them when needed?

- No, not at all
- Yes, but not completely
- Yes, complete information was provided

Other (please specify)

4. Did facilitators focus on body acceptance and love during the mindful movement and direct women to actively forgive themselves and be compassionate to parts they do not like as much?

- No, not at all
- Yes, but not completely
- Yes, complete information was provided

Other (please specify)

5. Did participants find a partner and go through the How Am I Doing? MAMAS Self-Assessment with one another, looking for places where they have similar struggles and others where they have differences?

- ◯ No, not at all
- ◯ Yes, but not completely
- ◯ Yes, complete information was provided

Other (please specify)

Self-Compassion (20 Minutes)

6. Did facilitators talk about the concept of self-compassion and self-forgiveness and share that having compassion for ourselves and our shortcomings makes us healthier, happier, and more resilient?

- No, not at all
- Yes, but not completely
- Yes, complete information was provided

Other (please specify)

7. Did facilitators lead the exercise by Kristin Neff allowing women to respond to the questions and challenge their beliefs that being harsh and self critical is what makes them better versus being more loving or compassionate toward the self?

- No, not at all
- Yes, but not completely
- Yes, complete information was provided

Other (please specify)

8. During the self compassion exercise did facilitators specifically bring up women's bodies and point out the handouts this week on how body obsession and how being mean to ourselves about our bodies typically does not result in healthier eating?

- No, not at all
- Yes, but not completely
- Yes, complete information was provided

Other (please specify)

9. Did facilitators lead a self-compassion mindfulness practice (beginning with an imaginary friend who is unconditionally loving, accepting, kind and compassionate toward them, then seeing themselves from this point of view)?

- No, not at all
- Yes, but not completely
- Yes, complete information was provided

Other (please specify)

10. Did facilitators lead a discussion about participants’ experience during this self-compassion practice and discuss how self-compassion can then be extended to other people – family members, partners, children etc.?

- No, not at all
- Yes, but not completely
- Yes, complete information was provided

Other (please specify)

Pot Luck (60 Minutes)

11. Did facilitators prepare the group for the pot luck by briefly reviewing the key guidelines and give participants time to briefly say something about how and why they chose the foods to bring for the pot luck?

- No, not at all
- Yes, but not completely
- Yes, complete information was provided

Other (please specify)

12. Did facilitators remind participants that they should go back for seconds of at least one food, and that they should leave food on their plates?

- No, not at all
- Yes, but not completely
- Yes, complete information was provided

Other (please specify)

13. Did facilitators lead a mini-meditation before people choose their food and ask them to use the 10 point Hunger Scale before they begin, to be aware of where their hunger is and “how they know"?

- No, not at all
- Yes, but not completely
- Yes, complete information was provided

Other (please specify)

14. Did facilitators direct the group to eat mindfully with the first half of the meal in silence, stopping people at least once during the silent period to bring awareness of their hunger on the 10-point hunger scale and fullness on the 10-point fullness scale?

- ◯ No, not at all
- ◯ Yes, but not completely
- ◯ Yes, complete information was provided

Other (please specify)

15. Did instructors indicate that it is okay to talk about half way through the meal, reminding them to do so mindfully?

- ◯ No, not at all
- ◯ Yes, but not completely
- ◯ Yes, complete information was provided

Other (please specify)

Discussion of Mindful Eating Experience (20 minutes)

16. Did facilitators lead a discussion of the mindful eating experience and ask how people did at paying attention to their hunger and satiety levels and how the social environment impacted them?

- ○ No, not at all
- ○ Yes, but not completely
- ○ Yes, complete information was provided

Other (please specify)

17. Did they discuss what this exercise might mean in terms of applicability to daily eating and to weight loss?

- ○ No, not at all
- ○ Yes, but not completely
- ○ Yes, complete information was provided

Other (please specify)

Review Commitments, Homework & Closing (15 minutes)

18. Did facilitators review the three commitments and add self-compassion to their commitments asking women if there is anything they can commit to doing this week that would be kind to themselves?

- No, not at all
- Yes, but not completely
- Yes, complete information was provided

Other (please specify)

19. Did facilitators review the homework assignments for the upcoming week?

- No, not at all
- Yes, but not completely
- Yes, complete information was provided

Other (please specify)

20. Was there a closing silent sitting practice that ended with a loving-kindness meditation where the following phrases are repeated internally:
• May I be safe.
• May I be happy.
• May I be healthy and strong.
• May I live with ease.

(Then repeating the intention again this time replacing "I" with "we")?

- No, not at all
- Yes, but not completely
- Yes, complete information was provided

Other (please specify)

1. Which audio recording are you coding? (please use the exact title from the Dropbox audio file, e.g. May 8 2012 Session 1)

Movement and Check-In (30 Minutes)

2. Did facilitators ask participants to turn in their Daily Record and if they did not complete or bring their Daily Record, did they give them a few moments to complete it from memory?

- ◯ No, not at all
- ◯ Yes, but not completely
- ◯ Yes, complete time was given

Other (please specify)

3. During the Mindful Movement series did instructors use less facilitation this week and see if participants can do the series from memory and focus on ways that these movements can be integrated into everyday life, (e.g. at a desk, doing dishes, laundry, when the baby comes etc.) and encourage women to begin to integrate these stretches and movements into their everyday life?

- ◯ .No, not at all
- ◯ Yes, but not completely
- ◯ Yes, complete information was provided

Other (please specify)

4. Did women go over the Self-Reflection Worksheet with a partner and check in about how they are doing with the Three Commitments?

- ◯ No, not at all
- ◯ Yes, but not completely
- ◯ Yes, complete information was provided

Other (please specify)

5. Did facilitators lead a discussion about the participants' progress, challenges, questions and concerns, and brainstorm for integration into everyday life (making sure each person gets a chance to talk about successes and challenges)?

- No, not at all
- Yes, but not completely
- Yes, complete information was provided

Other (please specify)

Mindful Eating, Review of Concepts & Broadening Food Repertoire (20 minutes)

6. Did facilitators ask participants to state the FIVE elements of mindful eating that were talked about during this training:

1. Mindful Awareness of Hunger
2. Mindful Awareness of Fullness (remember this is different from hunger!)
3. Mindful Awareness of Taste Satiety, stopping instead ofjfsdahasing the Flavor,
4. Mindful Awareness of Taste Satisfaction –stopping when a level of satisfaction or enjoyment is reached.
5. Mindful Awareness of Food Choices

And did they go over what each one means, paying special attention to using examples that help people incorporate them into their everyday lives?

- ◯ No, not at all
- ◯ Yes, but not completely
- ◯ Yes, complete information was provided

Other (please specify)

7. Did instructors lead a mindfulness eating exercise utilizing all five elements previously discuss with fresh, healthy, and unusual foods to broaden their repertoire and encourage participants to choose foods that they do not normally eat? (This exercise should be done in silence)

- ◯ No, not at all
- ◯ Yes, but not completely
- ◯ Yes, complete information was provided

Other (please specify)

8. Did instructors lead a discussion about their experiences during the mindful eating exercise making sure each participant shares something? Did they discuss carrying an insulated lunchbox around so that healthy foods of their choice are always available to them?

- No, not at all
- Yes, but not completely
- Yes, complete information was provided

Other (please specify)

Mindful Awareness in Everyday Life (30 minutes)

9. Did facilitators lead a mindful awareness exercise about bringing mindful awareness into everyday life talking about mindfulness with their baby as well as with relationships with partners, family members, co-workers, and strangers?

- ○ No, not at all
- ○ Yes, but not completely
- ○ Yes, complete information was provided

Other (please specify)

10. Did facilitators have participants list the elements of mindfulness and how they might be used in difficult moments in relationships? Did they write their answers on the board and embellish what they've already said, using the categories below:
-Awareness
-Acceptance
-Allowing/Letting Be
-Present-Moment Focus
-Beginners Mind/Curiosity
-Compassion

- ○ No, not at all
- ○ Yes, but not completely
- ○ Yes, complete information was provided

Other (please specify)

11. Did instructors lead a mindful decision making exercise having participants think of a difficult decision they need to make and have them notice any body sensations, feelings or emotions, thoughts, and intuitions about it?

- No, not at all
- Yes, but not completely
- Yes, complete information was provided

Other (please specify)

12. After the mindfulness exercise did participants share with a partner what they learned from their internal inquiry and end with stating the decision to their problem with 'I want…'? After this did they come together as a group to discuss the exercise?

- No, not at all
- Yes, but not completely
- Yes, complete information was provided

Other (please specify)

13. Did facilitators discuss how to bring a mindfulness practice into their everyday life and give examples such as taking a mindful shower or bath (telling them to be present through the whole shower and focusing on the body sensations, emotions, thoughts, and senses.) Did they explain that mindful awareness can be brought to almost any daily activity?

- No, not at all
- Yes, but not completely
- Yes, complete information was provided

Other (please specify)

14. Did facilitators go around the room and ask participants to suggest an activity that could be done in a mindful way and write them on the board?

- No, not at all
- Yes, but not completely
- Yes, complete information was provided

Other (please specify)

15. Did participants review the Friendly Tips for Mindfulness Practice handout together and add new ideas as they come up?

- No, not at all
- Yes, but not completely
- Yes, complete information was provided

Other (please specify)

Mindful Awareness Integrated Practice (20 minutes)

16. Did participants write down their answers to the following questions:

1) What might get in the way of you being able to keep up with the mindful eating, Eat More/Eat Less and moving your body?

2) What might help you keep doing these practices, or practicing them even more?

3) How realistic are the things that might help – in other words, which ones are you most likely to be able to do?"

- ◯ No, not at all
- ◯ Yes, but not completely
- ◯ Yes, complete information was provided

Other (please specify)

17. Did participants break into groups of three and talk about what they thought might get in the way of using the mindful practices and brainstorm solutions together? Were they encouraged to asses the realisticness of the solutions and choose a few to implement?

- ◯ No, not at all
- ◯ Yes, but not completely
- ◯ Yes, complete information was provided

Other (please specify)

18. Did participants come back to the large group and report what they found and did facilitators write themes on the board, including obstacles?

- ◯ No, not at all
- ◯ Yes, but not completely
- ◯ Yes, complete information was provided

Other (please specify)

Behavioral Commitments and Closing (10 minutes)

19. Did facilitators review the three commitments (Mindful eating, move my body, and breath)?

- ○ No, not at all
- ○ Yes, but not completely
- ○ Yes, complete information was provided

Other (please specify)

[ ]

20. Did facilitators review the "homework" and things that they can do for the rest of their pregnancy regarding the three commitments?

- ○ No, not at all
- ○ Yes, but not completely
- ○ Yes, complete information was provided

Other (please specify)

[ ]

21. Was the closing done in a circle, with the words below spoken out loud, together, while looking at one another using a call and response technique (facilitators say one line, then participants repeat it)?

• May I be safe.
• May I be happy.
• May I be healthy and strong.
• May I live with ease.

• May we be safe.
• May we be happy.
• May we be healthy and strong.
• May we live with ease.

- ○ No, not at all
- ○ Yes, but not completely
- ○ Yes, complete information was provided

Other (please specify)

[ ]
