# Supplementary material for: The mindful moms training: development of a mindfulness-based intervention to reduce stress and overeating during pregnancy
Source: BMC Pregnancy Childbirth. 2018 Jun 1;18:201. doi: 10.1186/s12884-018-1757-6 (PMC5984812; doi:10.1186/s12884-018-1757-6)
Supplement: Supplementary file 3 — MAMAS Study Final Evaluation Questionnaire. (PDF 71 kb) [file 12884_2018_1757_MOESM3_ESM.pdf]

m mas

Now that you have completed the Mindful Moms program, we would like to know about your experience. Your feedback will help us to improve the classes so please answer the questions as honestly as you can. We will summarize the responses of all class members before sharing it with the teaching staff. The information that you tell us will not be linked with your name. Thank you for taking the time to complete the evaluation.

1. How many Mindful Moms classes have you attended? ___________________

2. How convenient is the class location?
   ☐ Very convenient ☐ Somewhat convenient ☐ Not at all convenient

3. How convenient is the day and time of the class?
   ☐ Very convenient ☐ Somewhat convenient ☐ Not at all convenient

4. Did you have enough opportunities to ask questions of the teachers during or after class?
   ☐ Yes ☐ No ☐ Not applicable; I did not have any questions.

5. What is the main reason you enrolled in the program? _____________________________
   ____

6. How satisfied are you with the program so far?
   ☐ Very satisfied ☐ Satisfied ☐ Dissatisfied ☐ Very dissatisfied

7. Please list two things you like about the program.
   1. ____
   2. ____

8. Please list two things you dislike about the program.
   1. ____
   2. ____

9. If there was one thing you could change about the program, what would it be? ______________
   ____
   ____
   ____
   ____

10. How useful was learning to meditate, or do Mindful Awareness of Breathing?

☐ Very useful ☐ Useful ☐ Not very useful ☐ Not at all useful

a. How often have you used this tool over the past week?

☐ Did not use ☐ A few times ☐ Once a day on most days ☐ Several times on most days

b. Comments: ____

11. How useful was learning Mindful Movement – in other words, mindful walking or yoga?

☐ Very useful ☐ Useful ☐ Not very useful ☐ Not at all useful

a. How often have you used this tool over the past week?

☐ Did not use ☐ A few times ☐ Once a day on most days ☐ Several times on most days

b. Comments: ____

12. How useful was learning Mindful Eating?

☐ Very useful ☐ Useful ☐ Not very useful ☐ Not at all useful

a. How often have you used this tool over the past week?

☐ Did not use ☐ A few times ☐ Once a day on most days ☐ Several times on most days

b. Comments:____

13. How useful was the eating and movement advice for outside of class (like, the three basic commitments and tips for eating healthy and movement)?

☐ Very useful ☐ Useful ☐ Not very useful ☐ Not at all useful

a. Comments:____

In your own words, please explain what mindfulness is.

____

____

____

14. How well would you say you understand the idea of acceptance?

☐ Very well ☐ Somewhat well ☐ A little ☐ Not at all

15. Have you used any of the skills you learned in Mindful Moms outside of class?

☐ No

☐ Yes. Please tell us which skill you used and how you used it: ______________________

____

____

16. On average, how much time outside of class have you spent practicing these new skills each week?

________________ minutes or hours

(Please circle one)

17. Is there anything else you would like to tell us about your experience so far?

____

____

____

____
